# TDP43 and hnRNP K Regulate Alternative Splicing of DNAJC5

**DOI:** 10.1002/cbin.70158

**Published:** 2026-04-15

**Authors:** Helder Y. Nagasse, Ellen K. Okuda, Patricia P. Coltri

**Affiliations:** ^1^ Department of Cell and Developmental Biology, Institute of Biomedical Sciences University of São Paulo São Paulo São Paulo Brazil; ^2^ Institute of Molecular Biosciences Goethe University Frankfurt am Main Hesse Germany; ^3^ IMPRS on Cellular Biophysics Frankfurt am Main Hesse Germany

**Keywords:** alternative splicing, amyotrophic lateral sclerosis, DNAJC5 protein, HNRNPK protein, TARDBP protein

## Abstract

Alternative splicing is a finely regulated process which defines the final maturation of pre‐mRNAs. Modulation of trans‐acting spliceosome proteins changes specific patterns of splicing and contributes to the development of diseases. During Amyotrophic Lateral Sclerosis (ALS) disease progression, loss of nuclear trans‐acting splicing protein TDP43 leads to accumulation of cryptic exons in mRNAs, which inhibits expression of proteins and aggravates the disease. One of the affected genes is *DNAJC5*, which codes for a protein responsible for clearance of misfolded proteins in the cytoplasm. We first observed that TDP43 knockdown regulates DNAJC5 transcript splicing. A similar phenotype was observed upon hnRNP K knockdown. We hypothesized canonical splicing of DNAJC5 is dependent on the activity of both TDP43 and hnRNP K. Our results confirmed TDP43 and hnRNP K interaction is dependent on RNA. We also confirmed that DNAJC5 canonical splicing is dependent on its internal TDP43 and hnRNP K binding sites. Taken together, our work enrolls both TDP43 and hnRNP K on splicing regulation of DNAJC5 transcript, affecting activity of the protein encoded by *DNAJC5* on endosomal traffic. As a result, activity of both TDP43 and hnRNP K and their association are important for ALS progression.

## 1 Introduction

Amyotrophic Lateral Sclerosis (ALS) is a neurodegenerative disorder which affects upper and lower motor neurons (Grad et al. 2017). Degeneration of motor neurons leads to loss of their connection with muscles and consequently causes paralysis and respiratory failure. Along the progression of the disease, chronic oxidative stress and translocation of nuclear RNA‐binding proteins to the cytoplasm are observed (Ratti et al. 2020). TDP43 is an essential nuclear RNA‐binding protein translocated to the cytoplasm during ALS progression (Cairns et al. 2007; Oiwa et al. 2023). TDP43 has important roles in RNA metabolism, and can regulate transcription, alternative splicing, translation, and miRNA maturation (Buratti 2001; Kawahara and Mieda‐Sato 2012; Morera et al. 2019; Neelagandan et al. 2019).

Structurally, TDP43 has two RNA‐binding motifs, which recognize UG‐rich regions in pre‐mRNAs, and a C‐terminal domain enriched with glutamine and asparagine residues (Q/N) (Prasad et al. 2019). The Q/N‐rich repeats have intrinsically disordered regions (IDR) and facilitate protein–protein or RNA–protein interactions, allowing for protein oligomerization and aggregation (Hallegger et al. 2021). Upon oxidative stress, TDP43 is translocated from the nucleus to the cytoplasm, where it oligomerizes (Winton et al. 2008) and triggers the assembly of cytoplasmic stress granules, a non‐membrane organelle (Wolozin and Ivanov 2019). This cytoplasmic retention blocks TDP43 from performing its nuclear functions and impairs cellular homeostasis (Gasset‐Rosa et al. 2019).

A critically impaired process during TDP43 translocation is alternative splicing, a highly regulated co‐transcriptional process catalyzed by the spliceosome (Bonnal et al. 2020). Pre‐mRNA splicing removes introns and joins exons into mature mRNA sequences. Alternative splicing catalyzes the reunion of different exons and parts of introns, generating transcripts that might be non‐functional or expand the proteomic diversity, generating different phenotypes. Definition of exons and introns is directly dependent on recognition of conserved splice sites on the borders between exons and introns. During this process, trans‐acting proteins can drive the machinery to the selection of different splice sites, generating different transcripts (Dreyfuss et al. 1993; Lee and Rio 2015; Mayeda and Krainer 1992). As a result, alternative transcripts can generate truncated proteins or, in some cases, transcripts might be degraded even before translation, through the nonsense‐mediated decay pathway (NMD) (Kapustin et al. 2011; Lee and Rio 2015).

TDP43 is known to associate with hnRNPs (Freibaum et al. 2010) and to repress the inclusion of cryptic exons into mature transcripts during splicing (Ling et al. 2015). hnRNPs are a class of 24 conserved ribonucleoproteins (Geuens et al. 2016) mainly concentrated in the nucleus, with essential roles in transcription, alternative splicing, and polyadenylation regulation (Krecic and Swanson 1999; Mayeda and Krainer 1992; Veraldi et al. 2001). Similarly to TDP43, some hnRNPs can transit between the nucleus and cytoplasm, acting in nuclear export, mRNA localization and translation (Geuens et al. 2016). Removal of TDP43 from the nucleus affects the alternative splicing of genes involved with the oxidative stress control, neuro‐surveillance and axon guidance (Klim et al. 2019; Mohagheghi et al. 2016). As a result, loss of TDP43 contributes to ALS progression and neurodegeneration (R. H. Brown and Al‐Chalabi 2017; Klim et al. 2019). Alternative splicing of the Hsp70 chaperone co‐factor DNAJC5 is severely affected by TDP43 absence. *DNAJC5* codes for a co‐chaperone important to promote the correct folding of proteins and vesicle transport in ceroid neurons (Nosková et al. 2011; Russell et al. 1999). In addition, mutation or loss of DNAJC5 reduces the recruitment of the “misfolding association protein secretion pathway” (MAPS), an alternative pathway which allows the exit of misfolded cytoplasmic proteins from the cell in order to reduce neurotoxicity (Volkmar et al. 2016; Xu et al. 2018).

Changes in localization and abundance of these proteins lead to the development of several diseases, from cancer to neurodegenerative disorders (Brandão‐Teles et al. 2024; Purice and Taylor 2018). hnRNP K is a 51 kDa splicing factor which binds to enriched poly(C) sequences. Higher concentration of hnRNP K has been associated with development of frontotemporal dementia and different types of cancer (Y. Li et al. 2022; Moujalled et al. 2015). hnRNP K contains three KH motifs, responsible for RNA binding, and a RGG motif which exhibits liquid–liquid phase separation (LLPS) formation capacity (Ding et al. 2025; Valverde et al. 2008). Previous works reported TDP43 and hnRNP K interact during transcription of *NRF2*, which encodes for an oxidative stress response initiator (Moujalled et al. 2015). Upon mutation of TDP43, hnRNP K cannot be recruited as a transcription factor for *NRF2* transcription, disrupting the antioxidative response and worsening oxidative stress (Moujalled et al. 2017). We hypothesized TDP43 and hnRNP K could interact in the context of splicing. Our results indicated that TDP43 and hnRNP K are immunoprecipitated and share an RNA–dependent interaction within the nucleus, where both are co‐localized. Importantly, our results also showed generation of canonical DNAJC5 transcript responds directly to TDP43 and hnRNP K levels, reducing upon knockdown and increasing on overexpression. We also observed TDP43‐hnRNP K association is dependent on RNA and possibly occurs during transcription and splicing. In vitro reactions confirmed these proteins interact during DNAJC5 alternative splicing regulation. Functional experiments also showed that regulation of hnRNP K levels is important for DNAJC5 vesicle trafficking, suggesting that regulating the levels of TDP43 and hnRNP K can directly affect secretion of cytosolic misfolding proteins and contribute to disease progression.

## 2 Materials and Methods

### 2.1 Cloning

Overexpression plasmids for TDP43 and hnRNP K were constructed with pFLAG‐CMV3 vector (Sigma), which expresses a N‐terminal FLAG‐tagged protein. TDP43 and hnRNP K coding sequences were PCR‐amplified following subcloning in pFLAG‐CMV3 vector using specific primers, flanking restriction enzyme sites for HindIII and XbaI for TDP43, and EcoRI and EcoRV for hnRNP K, were added for cloning (listed in Table 1). Site‐directed mutagenesis in TDP43 sequence was performed upon pGEM‐T‐TDP43 plasmid using Q5 site‐directed mutagenesis kit (NEB) and oligonucleotides for Q331K and M337V mutations (listed in Table 1), generating pFLAG‐TDP43^Q331K^ and pFLAG‐TDP43^M337V^. CRISPR‐Cas9 plasmids were constructed using the pX330 vector (Etoc et al. 2016). RNA guides were designed using software GreenListed 2.0 (Panda et al. 2017) (listed in Table 1). After oligo phosphorylation and annealing, the duplex was subcloned in the pX330 plasmid vector, generating pX330‐g1TDP43 and pX330+g1hnRNPK. pDsRed‐hnRNP K was generated upon cloning of hnRNP K into pDsRed (Invitrogen). The DNAJC5 mini‐gene constructs were generated with the amplification of a 735 bp region comprising the genomic region encompassing exons 4 and 5 of DNAJC5. The PCR product was cloned in pGEM‐T‐easy (Promega) and sequenced to confirm integrity. In addition, Q5 site‐directed mutagenesis kit (NEB) was used to delete predicted binding sites of TDP43 and hnRNP K generating pGEM‐DNAJC5 dUG and pGEM‐DNAJC5 dC, respectively.

**TABLE 1:** Sequence of primers used in cloning, CRISPR, mutagenesis, and qPCR experiments.

Primer	Sequence (5′− 3′)
Cloning TDP43 forward	GCCAAGCTTATGTCTGAATATATTCGGGT
Cloning TDP43 reverse	GTATCTAGACTACATTCCCCAGCCAGAAG
Cloning hnRNP K forward	AGCGAATTCAATGGAAACTGAACAGCCAGAAGAAAC
Cloning hnRNP K reverse	CTCGATATCTTAGAATCCTTCAACATCTGCATAC
Mutagenesis primer TDP43 Q331K forward	GGCAGCACTAAAGAGCAGTTGG
Mutagenesis primer TDP43 Q331K reverse	TGGGCGGCAGCCATCATG
Mutagenesis primer TDP43 M337V forward	TTGGGGTATGGTGGGCATGTTAG
Mutagenesis primer TDP43 M337V reverse	CTGCTCTGTAGTGCTGCC
CRISPR guide 1 TDP43 pair 1	CACCGGAAATACCATCGGAAGACGATGG
CRISPR guide 1 TDP43 pair 2	AAACCCCATCGTCTTCCGATGGTATTTC
CRISPR guide 1 hnRNP K pair 1	CACCGTAAAATCAAAGAACTTCGAGAGG
CRISPR guide 1 hnRNP K pair 2	AAACCCCTCTCGAAGTTCTTTGATTTTA
qPCR TDP43 forward	CCATCGGAAGACGATGGGAC
qPCR TDP43 reverse	TGGGGCATGCAGAATTCCTT
qPCR hnRNP K forward	GGACGTGCACAGCCTTATGA
qPCR hnRNP K reverse	CGCGACGGTCATCAAACATC
qPCR β‐actin forward	ACCTTCTACAATGAGCTGCG
qPCR β‐actin reverse	CCTGGATAGCAACGTACATGG
qPCR snRNA U6 forward	CTCGCTTCGGCAGCACATATAC
qPCR snRNA U6 reverse	GGAACGCTTCACGAATTTGCGTG
qPCR DNAJC5 forward	GACGAGAGGGAGGCCACA
qPCR DNAJC5cpt forward	ACGAGAGGGGAGGGCACTGAC
qPCR DNAJC5 and DNAJC5cpt reverse	AGTTGAACCCGTCAGTGTGGTAG
qPCR ATG4B forward	CGCTGTGGGGTTTTTCTGTA
qPCR ATG4B reverse	CACCTAGGGACAGGTTCAGG
qPCR ATG4Bcpt forward	ATCGCTGTGGCCACCTGAG
qPCR ATG4Bcpt reverse	TACAGAAAAACCCCACGCTCAC
DNAJC5 exon 4 forward	TCTAAGCTTGCCCTGTTTGTCTTCTGCGGC
DNAJC5 exon 5 reverse	ACTGGATCCTTAGTTGAACCCGTCAGTGTGG
Mutagenesis primer DNAJC5 mini‐gene dUG forward	AGGGCCCGTGTGTG
Mutagenesis primer DNAJC5 mini‐gene dUG reverse	CACTCACCCCTCTCG
Mutagenesis primer DNAJC5 mini‐gene dC forward	GGGCAGAGCCAGAATGGGC
Mutagenesis primer DNAJC5 mini‐gene dC reverse	CGGGCCCTGGGGCG

### 2.2 Cell Culture and Transfection

HEK‐293FT cells (RRID:CVCL_6911), derived from human embryonic kidney, were obtained from Invitrogen (catalog# R70007) and HeLa cells derived from human female cervical cancer (ATCC Cat# CCL‐2, RRID:CVCL_0030) were cultured in 100 mm^2^ plates at 37°C and 5% CO2, in DMEM high glucose (Thermo) with the addition of 10% fetal bovine serum, 100 U/mL penicillin (Hyclone), 100 µg/mL streptomycin (Hyclone), and 0.25 µg/mL amphotericin B (Hyclone). Cultures were checked for contamination at each passage. To overexpress TDP43 and hnRNP K, non‐stable transfections of pFLAG+TDP43 and pFLAG+hnRNP K plasmids were performed using Lipofectamine 2000 (Thermo), according to manufacturer's instructions. As a control, an empty pFLAG plasmid was also transfected. Cells were selected using 1000 µg/mL gentamicin and collected after trypsinization with diluted 10× trypsin solution, 0.5% EDTA‐trypsin (Thermo). For pX330 plasmids, cells were selected with puromycin at a concentration of 500 ng/mL. After growth, cells were subjected to clonal selection. For NMD inhibition, cells were treated with cycloheximide (Biomol International LP) at a concentration of 100 µg/mL for 6 h (Ishigaki et al. 2001; Z. Li et al. 2017).

### 2.3 RNA Extraction and cDNA Synthesis

Cells were collected upon trypsinization and washed with a PBS buffer. Pellets were subjected to RNA extraction using Trizol (Thermo), according to the manufacturer's instructions. Total RNAs were precipitated with 3 M sodium acetate pH 5.2 and ethanol. cDNA synthesis was performed with the SuperScript IV Reverse Transcriptase kit (Thermo) and 2.5 µM random primers. Briefly, 5 µg of RNAs were used per reaction and cDNA synthesis was carried out after incubation at 25°C for 5 min, 50°C for 60 min and 70°C for 15 min.

### 2.4 Real‐Time RT‐qPCR

RT‐qPCR reactions were performed in 96‐well plates, with experimental triplicates for each group, using Step One Plus equipment (Applied Biosystems RRID:SCR_025855). One hundred nanograms of cDNA, 3.2 pM of forward and reverse primers (listed in Table 1), and SYBR Green (Fermentas) were used in a final volume of 12 µL per reaction. *ACTB* gene was used as an endogenous normalizer, in graphics it is shown as “β‐actin”. For in vitro splicing assays, *RNU6B* was used as an endogenous normalizer. The relative expression of the transcripts was analyzed using the 2^−ΔΔCt^ method (Livak & Schmittgen, 2001). The graphs were created using the GraphPad PRISM program v5.01 (RRID:SCR_000306).

### 2.5 In Vitro Splicing

Nuclear extracts were prepared from HeLa cells (ATCC Cat# CCL‐2, RRID:CVCL_0030), grown as indicated above until 80%–90% confluence, according to previously reported protocol (Nilsen 2013). In vitro transcriptions were performed using T7 RNA polymerase (Thermo) and linearized DNA plasmids pGEMte+DNAJC5, pGEMte+DNAJC5dUG, and pGEMte+DNAJC5dC, previously purified with phenol:chloroform. After that, in vitro splicing reactions were prepared by adding a 1 × splicing mix (20 mM creatine phosphate, 2 mM ATP, 30 mM KCl, 4.2 mM MgCl_2_, 4 mM DTT, 16 mM HEPES, 3% PEG 8000), 4 µL HeLa nuclear extract, 20 U RNAse inhibitor and 400 ηg of in vitro transcribed RNAs. Reactions were incubated at 28°C for 30 min, and the products were precipitated with phenol:chloroform followed by ethanol precipitation. The products of in vitro splicing were analyzed by RT‐qPCR.

### 2.6 Protein Extraction and Western Blot

Protein extraction was performed by adding 25 µL RIPA buffer per mg of cells (50 mM sodium phosphate, 5 mM NaCl, 1 mM EDTA, 0.5 mM EGTA, 10 mM Hepes pH 7.4, 10% Triton X‐100, 2 µL protease inhibitor cocktail (Amresco) and 0.25 µM DTT). Samples were quantified using Bradford method (Green and Sambrook 2012). Approximately 20 µg of proteins were separated by 10% polyacrylamide gel electrophoresis (SDS‐PAGE) at 100 V for 2 h in the Tris‐glycine buffer. Proteins were transferred to a nitrocellulose membrane (Thermo), using a wet transfer buffer at 80 mA for 3 h 30 min. The membrane was blocked in 3% BSA solution in TBS‐T (10 mM Tris‐base, 150 mM NaCl and 0.01% Tween‐20) at 4°C for 16–18 h and then incubated with the primary antibody in TBS‐T solution with 0.1% BSA for 16–18 h at 4°C. Anti‐TDP43 (Proteintech Cat# 10782‐2‐AP, RRID:AB_615042) was used at a dilution of 1:10,000, anti‐FLAG M2 (Sigma‐Aldrich Cat# F3165, RRID:AB_259529) at dilution of 1:1000. The anti‐β‐actin antibody (Sigma‐Aldrich Cat# A1978, RRID:AB_476692) at a dilution of 1:10,000 was used as an endogenous control. Following that, three 20‐min washes were performed in TBS‐T buffer and the membranes were incubated for 1 h with Secondary Antibody anti‐Mouse (Sigma‐Aldrich Cat# A9044, RRID:AB_258431) or Secondary Antibody anti‐Rabbit IgG (Sigma‐Aldrich Cat# A0545, RRID:AB_257896) in dilution 1:10,000. Proteins were detected by chemiluminescence with the ECL Western Blotting Substrate Kit (Thermo). Band densitometry was performed using the ImageJ software (RRID:SCR_002285).

### 2.7 Immunoprecipitation

For immunoprecipitation, HEK‐FLAG and HEK‐hnRNP K cell pellets were resuspended in buffer A (10 mM KCl, 1.5 mM MgCl2, 20 mM HEPES pH 7.5) and lysed with the homogenizer (Douncer). Approximately 1 mL of buffer was used per gram of cell pellet. Samples were quantified using the Bradford method (Green and Sambrook 2012). After quantification, 40 µg of cell lysate was collected (Input) and the remainder was used for immunoprecipitation. RNAase treatment was performed with addition of 0.01 µg/µL of RNase A (Invitrogen). To perform immunoprecipitation, the cell lysate was incubated with 70 µg protein A‐sepharose resin (GE Healthcare), previously coupled to anti‐FLAG M2 (Sigma‐Aldrich Cat# F3165, RRID:AB_259529) and pre‐equilibrated in TBS buffer. For RNA‐Immunoprecipitation the resin was previously blocked with 0.01 µg/mL of glycogen (Thermo) and 0.01 µg/mL of yeast tRNA (Ambion). After 5 h of incubation under shaking at 4°C, the sample was centrifuged at 10,000 rcf for 1 min and the supernatant was collected (flow‐through). Then, three washes were performed using 5 CV of buffer A, followed by centrifugations for 1 min at 10,000 rcf. Then, two elutions were carried out with a solution containing 1.5 µg of FLAG‐peptide in 100 µL TBS, for 40 min. To observe the association of TDP43 and hnRNP K, the input, flow‐through, and first and second elution samples were separated by electrophoresis in 10% SDS‐PAGE and a western blot was performed using anti‐TDP43 (Proteintech Cat# 10782‐2‐AP, RRID:AB_615042) and anti‐FLAG M2 (Sigma‐Aldrich Cat# F3165, RRID:AB_259529) antibodies. The bands were quantified using the ImageJ program (RRID:SCR_002285). For RNA‐Immunoprecipitation, input and elution samples were subjected to RNA extraction with Trizol protocol as described above, followed by cDNA synthesis and qPCR using primers DNAJC5 exon 4 forward and DNAJC5 exon 5 reverse.

### 2.8 Immunofluorescence

A total of 5 × 10^5^ cells/mL were transfected with pFLAG, or pFLAG+hnRNP K plasmid. Two days after transfection, cells were grown in a 6‐well plate, with 2 coverslips each. One day after plating, cells were treated with 0.5 mM sodium arsenite for 1 h (Wheeler et al. 2016), and embedded with 4% paraformaldehyde for 10 min. Then, coverslips were washed 3× with PBS, and incubated with 0.5% Triton‐X 100 in PBS for 10 min. Following that, cells were blocked with BSA 5% for 1 h, and incubated with anti‐TDP43 (Proteintech Cat# 10782‐2‐AP, RRID:AB_615042) or anti‐RAB9A (Cell Signaling Technology Cat# 5118, RRID:AB_10621426) at dilution 1:200 overnight. After that, the samples were washed with PBS and incubated with fluorescent rabbit secondary antibody Alexa Fluor 488 (Thermo Fisher Scientific Cat# A‐11034, RRID:AB_2576217) at dilution of 1:500 for 1 h. The coverslips were washed again with PBS and incubated with 300 ηM DAPI (Sigma) for 5 min. Following that, three washes with PBS were performed, and finally they were glued to the slide with 5 µL of Prolong gold antifade (Thermo). Cell photos were captured in Zeiss Axio Imager M2 (RRID:SCR_024706), captured in ZEN Blue software (RRID:SCR_013672) and 630× zoom in. Images were analyzed in Fiji software (RRID:SCR_002285).

### 2.9 Image Analysis

Using Fiji software ImageJ (RRID:SCR_002285) v.1.54, images had brightness and contrast adjusted equally for the analysis. We analyzed three fields from three different biological samples for each group. Plot profile analysis was carried out using a 7‐µm length line followed by measures of blue, red, and green intensities signals. Data was plotted using distance (microns) and gray scale at *y*‐axis. In the present work we show three plot profiles for each group.

### 2.10 Statistical Analysis

The results obtained from the RT‐qPCR and western blot experiments were analyzed using one‐way ANOVA considering three biologically independent groups, and Tukey's post hoc. Normality and homogeneity of variance were checked with GraphPad software (RRID:SCR_000306). Regarding the results of hnRNP K overexpression, a two‐tailed *t*‐test analysis was performed, with a significance level of 0.05. Normality was checked before the analysis with GraphPad software (RRID:SCR_000306). Finally, co‐immunoprecipitation and in vitro splicing results were analyzed by a one‐tailed test with a significance level of 0.05. Analysis was performed using GraphPad software (RRID:SCR_000306). For each experiment three biological replicates were analyzed.

### 2.11 In Silico Analysis

In a search for TDP43 partners, we performed an in silico analysis using the STRING database (RRID:SCR_005223). To generate a network of interactors, we constructed a STRING network where each edge represents a correlation of confidence. The minimum required interaction score for confidence was 0.900, calculated by the STRING algorithm. The following program was used as interaction sources: Textmining; Experiments; Database; Co‐expression; Neighborhood; Gene Fusion; and Co‐occurrence. Forty‐four nodes were marked. After GO enrichment, 18 proteins related to RNA splicing (GO:0008380) were highlighted. The TDP43 interactors and RNA splicing related proteins were compared by its combined score.

In search for RBPs motifs in DNAJC5 RNA sequence, we performed an in silico analysis with the BRIO software (Guarracino et al. 2021), using the sequence between DNAJC5 exons 4 and 5 as input. The minimum *p*‐value accepted was 0.05, calculated by the BRIO software algorithm. Regions next to hnRNP K and TDP43 motifs were plotted.

## 3 Results

### 3.1 TDP43 Affects Alternative Splicing of ALS‐Related Genes

Previous literature reported that TDP43 removal from the nucleus during ALS development increases the inclusion of cryptic exons (Ling et al. 2015). To observe how alternative splicing is regulated under different concentrations of TDP43, especially concerning the inclusion of cryptic exons, we initially knocked down TDP43 and observed splicing in two selected genes with cryptic exons frequently included on mRNAs during ALS (Supplementary Figure 1). In this work, we compared the canonical and alternative splicing isoforms of DNAJC5, a Hsp70 chaperone co‐factor, and ATG4B, an autophagy pathway regulator. Previous data reports that TDP43 regulates inclusion of ATG4B cryptic exon 10/11 and DNAJC5 cryptic exon 4/5 (Ling et al. 2015; Polymenidou et al. 2011; Schmidt et al. 2019; Torres et al. 2024; Xu et al. 2018). Specific primers to distinguish between the canonical and alternative isoforms were designed (Supplementary Figure 1), canonical isoforms are referred as their transcript name (DNAJC5 and ATG4B), and alternative isoforms are referred as DNAJC5cpt and ATG4Bcpt (Supplementary Figure 1).

**FIGURE 1:**
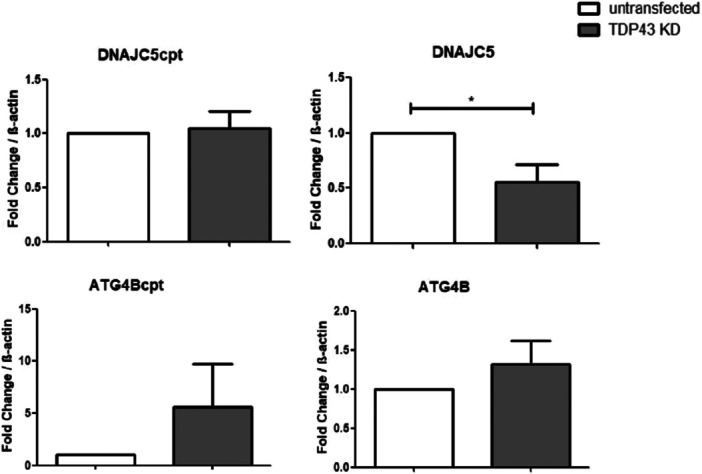
Quantification of isoforms of ALS‐related genes DNAJC5 and ATG4B. On the left, quantification of alternative isoforms and, on the right, is shown the quantification of canonical isoforms of the respective genes, upon TDP43 knockdown in HEK‐293T cells. Three independent samples of TDP43 knockdown cells (TDP43 KD) (gray bars) and untransfected cells (white bars) were compared. Results were normalized to β‐actin and the fold change was calculated as described in the methods. Three independent assays were performed. Statistics were performed with an independent two‐tailed *t*‐test. Error bars indicate the mean standard error. *p < 0.05.

To modulate the levels of TDP43, we edited the TDP43 coding sequence in HEK‐293T cells using the CRISPR‐Cas9 tool to knockdown its levels, generating TDP43 KD cells. Knockdown was confirmed by RT‐qPCR and western blot. TDP43 KD cells showed approximately 60% of regular TDP43 mRNA levels (Supplementary Figure 2A). Western blot analysis using cell extracts of TDP43 KD cells and untransfected HEK‐293T confirmed reduction on TDP43 protein levels (Supplementary Figure 2B). Gene edition was also confirmed using Sanger DNA sequencing reaction (Supplementary Figure 2C), by using DNA from edited TDP43 KD cells. We observed a mixed population of cells, where edited cells had a GG‐to‐CC mutation in position 60–61 of TDP43. Depletion of TDP43 is described to promote splicing of a longer isoform of DNAJC5 (Polymenidou et al. 2011), which contains the cryptic exon. We observed significantly reduced levels of canonical DNAJC5 (Figure 1), whereas DNAJC5cpt isoform did not show alteration upon use of TDP43‐KD cells. We reasoned that a greater amount of DNAJC5 cryptic isoform was degraded by nonsense‐mediated decay (NMD), which impeded us to detect this isoform. This was confirmed by NMD inhibition assays with cycloheximide. Upon cycloheximide treatment, increased levels of DNAJC5 and DNAJC5cpt were observed, suggesting that both isoforms are regulated by NMD (Supplementary Figure 3). This reinforces that TDP43 KD cells have more cryptic isoforms than initially assessed by qPCR. Consistently, levels of DNAJC5 canonical isoform were reduced, similar to the expected for the ALS phenotype. These results indicated that TDP43 is required to keep regular levels of canonical DNAJC5, and to control cryptic exon inclusion in this transcript (Figure 1). As for ATG4B, we observed a non‐significant increase in both isoforms, as expected by previous literature data, with no difference in the amount of canonical isoform (Figure 1).

**FIGURE 2:**
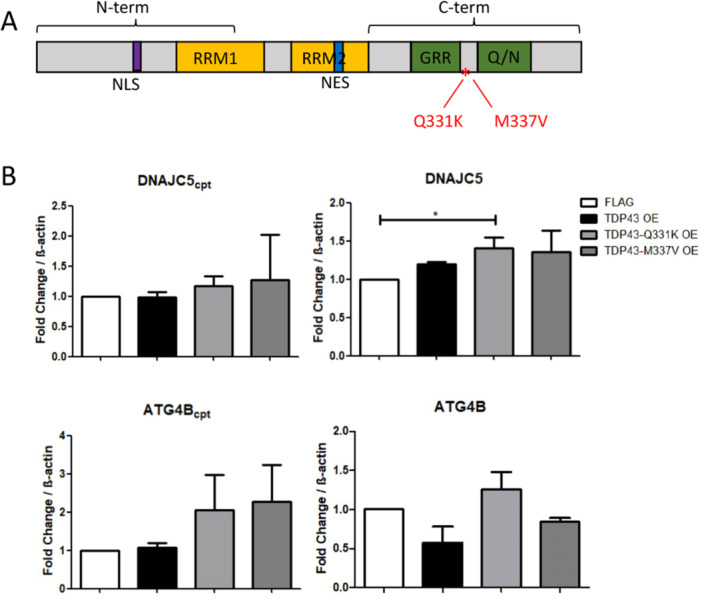
Overexpression analysis of wild type and mutant TDP43. (A) Representative scheme of the TDP43 amino acid sequence. The N‐terminal region has a nuclear localization site (NLS, purple box), followed by two RNA recognition domains (RRM1 and RRM2, yellow boxes). A nuclear export site (NES, blue box) is located within RRM2. The C‐terminal region has a glycine‐rich region (GRR, green box) and a glutamine and asparagine‐rich region (Q/N, green box). Residues Q331 and M337, where mutations were performed, are highlighted in red in the C‐terminal region. (B) Quantification of DNAJC5 and ATG4B alternative isoforms on the left, and canonical isoforms on the right, upon wild‐type TDP43 overexpression (TDP43 OE in black bars), TDP43^Q331K^ overexpression (TDP43^Q331K^ OE in light gray bars), and TDP43^M337V^ overexpression (TDP43^M337V^ OE in dark gray bars). FLAG cells were used as controls (white bars). Three independent samples were compared for each transcript. Results were normalized to β‐actin and the fold change value was calculated as described in the methods. Three independent assays were performed. Statistics was performed with a one‐way ANOVA and Tukey's post hoc using FLAG cells as a control. Error bars indicate the mean standard error. *p < 0.05.

We then asked whether a particular region of TDP43 could be important for its activity on splicing. TDP43 has two major structural domains. The N‐terminal has a nuclear localization signal (NLS) followed by two classical RNA‐binding motifs (RRM1 and RRM2). The C‐terminal region is formed by intrinsic disordered regions due to a glycine‐rich region (GRR) and a glutamine/asparagine‐rich region (Q/N) (Figure 2A). Importantly, more than 40 different mutations have already been reported for TDP43 C‐terminal region in familial ALS patients (Buratti 2015). We thus performed site‐directed mutagenesis in two C‐terminal sites of TDP43, Q331K, and M337V (Figure 2A). Similar levels of overexpression were reported for TDP43 and its mutated versions using the pFLAG system in HEK‐293T cells, as observed by RT‐qPCR and western blot (Supplementary Figure 4). A 2.5‐fold enrichment of TDP43 mRNA in HEK‐293T transfected cells, and an increase of 40%–50% on the protein levels confirmed overexpression (Supplementary Figure 4).

Upon wild‐type TDP43 overexpression, no significant differences on alternative splicing of DNAJC5 or ATG4B transcripts were observed (Figure 2B, black bars). We interpreted that an increase in wild‐type TDP43 expression does not lead to higher TDP43 occupancy on transcripts, therefore not changing the splicing pattern observed. A statistically significant increase was only observed on the canonical isoform of DNAJC5 upon TDP43^Q331K^ overexpression (Figure 2B). Importantly, this was not observed with wild‐type or TDP43^M337V^ overexpression, indicating the region comprising residue Q331 is critical for splicing regulation. A modest non statistically‐significant increase on ATG4Bcpt was also observed upon overexpression of TDP43^Q331K^ and TDP43^M337V^. TDP43 has been described to have a self‐regulatory system, capable of regulating its own levels if they are increased (Ayala et al. 2011). Previous literature data indicated this self‐regulation depends on the TDP43 C‐terminal region, near the region comprising residues 321–366 (Ayala et al. 2011). It is possible that mutations on this region have partially blocked the self‐regulatory characteristic of TDP43, thus making the mutants more resistant to self‐regulation, however, further evidence is required to confirm that in a cell‐based model (Ayala et al. 2011; Janssens et al. 2013; White et al. 2018). Presence of mutated TDP43^Q331K^ might compromise the function of TDP43 generating a splicing pattern similar to knockdown of TDP43 (Arnold et al. 2013). However, for some specific transcripts, TDP43^Q331K^ can enhance TDP43 function (Fratta et al. 2018). For example, TDP43^Q331K^ has been reported to increase exon skipping of Sortilin 1 and Eif4h (Fratta et al. 2018). We observed that overexpression of TDP43^Q331K^ increased canonical DNAJC5 transcript. Considering these results, we reasoned that wild‐type TDP43 could require additional factors to participate in the splicing regulation of this transcript, possibly associated with its C‐terminal region.

### 3.2 TDP43 and hnRNP K Interact in an RNA‐Dependent Manner

**FIGURE 3:**
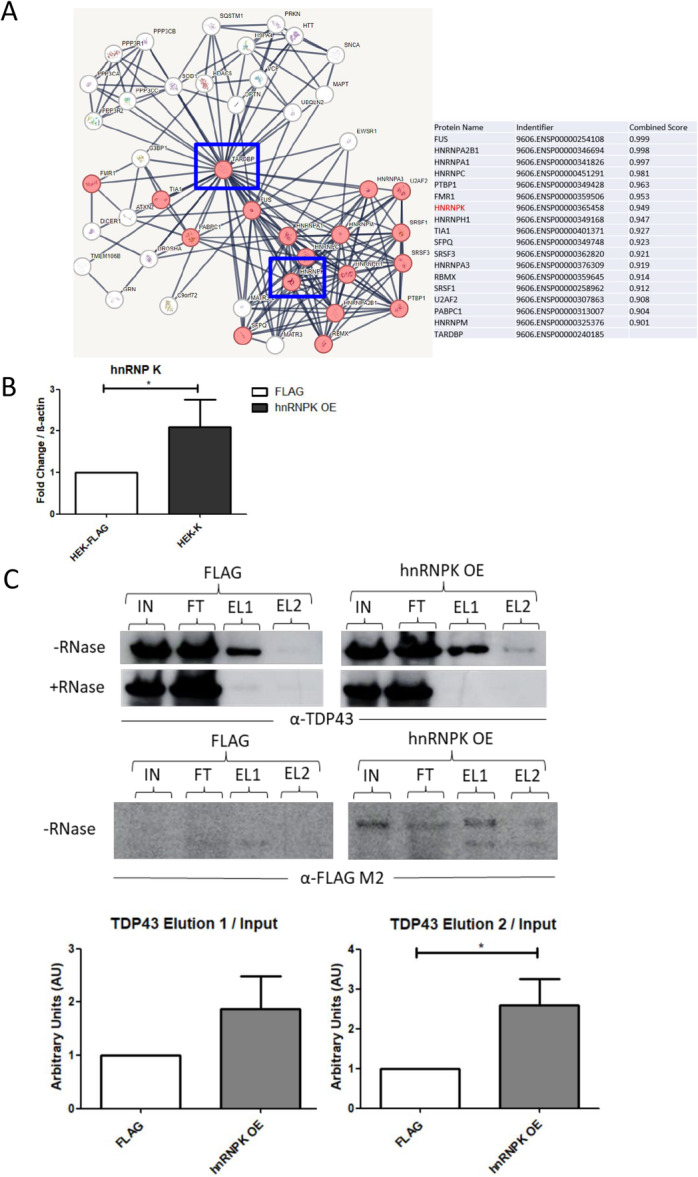
hnRNP K interacts with TDP43. (A) In silico analysis using STRING retrieved interactors of TDP43 generating a network. Data was collected using STRING software as described in the Methods. TDP43 and hnRNP K are highlighted in blue rectangles. The table on the right shows the higher combined scores between TDP43 and proteins involved in RNA splicing pathway. hnRNP K is highlighted in red. (B) RT‐qPCR reactions using control FLAG (white bars) and hnRNP K overexpression (hnRNP K OE) (black bars) samples. Fold change of hnRNP K was calculated using β‐actin as a normalizer. (C) Co‐immunoprecipitation assay of TDP43 and hnRNP K in HEK‐293T cells. Western blot using anti‐TDP43, anti‐FLAG and samples from FLAG (control) and hnRNP K OE cells. Input (IN), flow‐through (FT), and eluates 1 and 2 (EL 1 and EL2) were separated on SDS‐PAGE and transferred to membranes. The experiment was performed with and without addition of RNAseA to the input samples. Densitometry of the bands shown in eluate 1 and eluate 2 on FLAG and FLAG‐hnRNP K (anti‐TDP43) were compared and normalized with the respective inputs. Three independent assays were performed. Statistics was performed with a one‐way paired *t*‐test (*p < 0.05). Error bars indicate the mean standard error.

We hypothesized that the C‐terminal region of TDP43 could be essential to mediate interactions with other proteins during splicing. We performed an in silico analysis using STRING and filtered the results for TDP43 partners associated with splicing regulation (Figure 3A). Our analysis retrieved 18 proteins, including hnRNP K (Figure 3A). hnRNP K has also been described as a partner for TDP43 in previous mass spectrometry analysis (Freibaum et al. 2010). hnRNP K belongs to the hnRNP family of proteins and is already known to associate with promoter of *TARDBP1*, the coding gene for TDP43 (Hasegawa‐Ogawa and Okano 2021), also playing a role in the transcription of other genes (Moujalled et al. 2015). Recently, hnRNP K has been described to promote the generation of an additional alternative isoform of TDP43, with a non‐functional C‐terminal domain (Hasegawa‐Ogawa et al. 2025) concomitantly with full‐length TDP43. This isoform of TDP43 is not recognized by TDP43 antibodies because it lacks part of its C‐terminal domain (Hasegawa‐Ogawa et al. 2025). To address hnRNP K association with TDP43 in HEK‐293T cells, we performed co‐immunoprecipitation assays. By using the FLAG system, we overexpressed hnRNP K in HEK‐293T cells (Figure 3B). Total extract of FLAG‐hnRNP K cells (hnRNP K OE) was incubated with IgG beads coupled to anti‐FLAG antibody. Input, flowthrough, and elution fractions were separated on 10% SDS‐PAGE and immunoblots using anti‐TDP43 and anti‐FLAG were performed. After normalization by the input, the relative amounts of TDP43 found in eluate 2 were estimated to be ~2 times higher in hnRNP K OE cells than in FLAG cells (Figure 3C). Importantly, we performed the same assay with and without RNAseA added prior to the immunoprecipitation. Addition of RNAse abolished TDP43 association to hnRNP K, thus indicating that hnRNP K–TDP43 association is dependent on RNA (Figure 3C). Immunoblot using anti‐FLAG M2 was performed as control (Figure 3C). Taken together these results indicate TDP43 and hnRNP K interact in an RNA‐dependent manner. To address whether this interaction could occur in the context of RNA processing, we performed RNA extraction from input and elution fractions and searched for the DNAJC5 transcript by qPCR. RIP‐qPCR assay revealed increased levels of DNAJC5 RNA transcript associated with hnRNP K, as observed by the ratio of Ct's in elution and input samples (Supplementary Figure 5).

### 3.3 hnRNP K Can Affect Transcription and Alternative Splicing of ALS‐Related Genes

**FIGURE 4:**
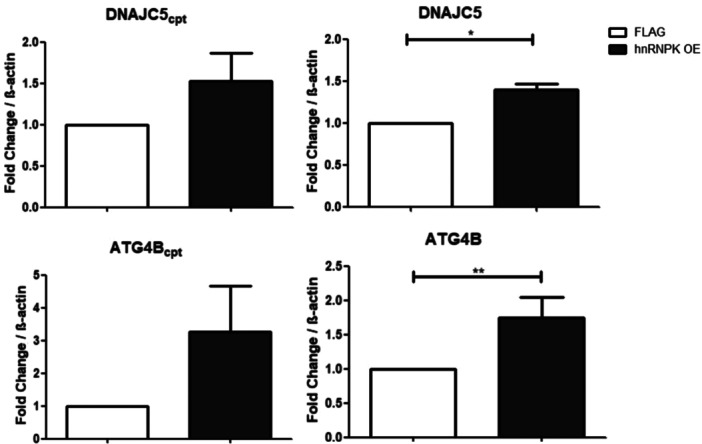
Quantification of DNAJC5 and ATG4B isoforms using hnRNP K overexpression cells (hnRNP K OE in black bars). Alternative isoforms shown on the left and canonical isoforms shown on the right. Three independent samples were compared for each transcript. Results were normalized to β‐actin and the fold change value was calculated as described in the methods. Three independent assays were performed. Statistics were performed with an independent two‐tailed *t*‐test. Error bars indicate the mean standard error. *p < 0.05 **p < 0.01.

**FIGURE 5:**
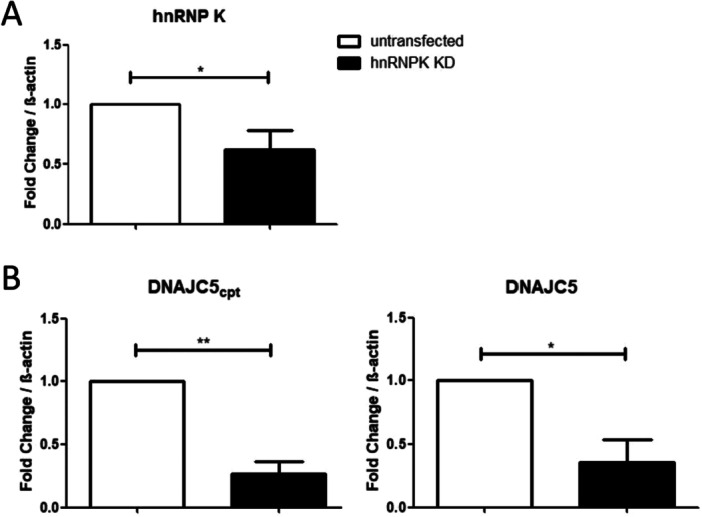
Analysis of DNAJC5 isoforms using hnRNP K knockdown cells (hnRNP K KD). (A) RT‐qPCR reactions using untransfected cells (white bars) and hnRNP K KD (black bars) HEK‐293T cells. Fold change of hnRNP K was calculated using β‐actin as a normalizer. (B) Quantification of DNAJC5 isoforms under hnRNP K KD. Three independent samples were compared. Results were normalized to β‐actin and the fold change value was calculated as described in the methods. Statistics were performed with an independent two‐tailed *t*‐test. Error bars indicate the mean standard error. *p < 0.05 **p < 0.01.

Given that TDP43 and hnRNP K can associate and share a nuclear localization during non‐pathological conditions (Ayala et al. 2008; Dejgaard et al. 1994), possibly through DNAJC5, we asked whether hnRNP K affects ATG4B and DNAJC5 splicing (Figure 4). Upon hnRNP K overexpression (Figure 3B), a significant increase on the canonical isoforms of transcripts ATG4B and DNAJC5 was observed (Figure 4, right panels). This result was expected, since previous data suggested that presence of hnRNP K is important for canonical splicing (Bampton et al. 2021). An increase in ATG4Bcpt and DNAJC5cpt was also observed with no statistical significance (Figure 4, left panels). This result suggested that hnRNP K can affect not only splicing but also transcription of DNAJC5. Importantly, hnRNP K has also been described as a global transcription factor in adenocarcinoma (Y. Li et al. 2022). We interpreted that hnRNP K affects transcription of DNAJC5 and ATG4B pre‐mRNAs, leading to an increase of both isoforms analyzed, canonical and cryptic. Consistently, when we performed hnRNP K knockdown using CRISPR‐Cas9 in HEK293T cells, reducing to ~40% of its mRNA levels (Figure 5A), reduced levels of DNAJC5 canonical and cryptical isoforms were observed (Figure 5B). Importantly, it does not discard a secondary effect of hnRNP K on alternative splicing of DNAJC5. Consistent with the hnRNP K OE profile, an increase of DNAJC5 canonical isoform was also observed upon TDP43^Q331K^ OE (see Figure 2B), reinforcing that both proteins possibly act together during splicing, and possibly through TDP43 C‐terminal. We interpreted these results as an indication that hnRNP K overexpression results in a phenotype that stimulates generation of the canonical isoforms of the transcripts, therefore the protein is necessary to maintain these isoforms.

### 3.4 TDP43 and hnRNP K Act on DNAJC5 Alternative Splicing

**FIGURE 6:**
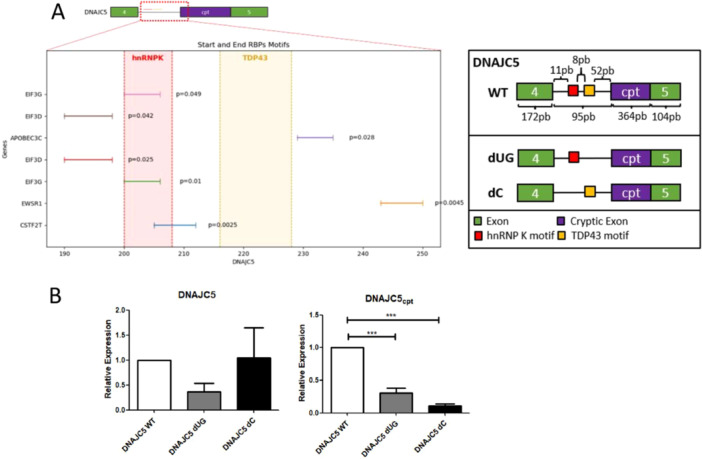
In vitro splicing assays confirm TDP43 and hnRNP K participate in DNAJC5 splicing. (A) On the left, predicted motifs for RBP binding along DNAJC5 sequence region spanning exon 4, intron, cryptic exon, and exon 5. The *x*‐axis is a zoom of the intronic region (red square). On the *y*‐axis, seven predicted RBPs bind around this region, as predicted by the BRIO software (Guarracino et al. 2021). The estimated confidence of the prediction shown by the *p*‐value is also indicated. In this region, there is a TDP43 binding motif, marked in yellow between nucleotides 216 and 228, and an hnRNP K binding motif, marked in red between nucleotides 200 and 208. On the right, schematic representation of DNAJC5 mini‐gene constructions, comprising the genomic region between exons 4 and 5. The exon 4, cryptic exon and exon 5 are represented by green‐ and purple‐colored boxes. TDP43 predicted poli‐UG binding site and hnRNP K predicted poli‐C binding site are represented in yellow and red boxes within this intron, respectively. Size of each motif and distance length are described in base pairs. Schemes of the generated mini‐genes with deletion of TDP43 binding motif (DNAJC5 dUG), and deletion of hnRNP K binding motif (DNAJC5 dC) are shown. (B) Relative levels of canonical DNAJC5 and cryptic DNAJC5cpt isoforms after in vitro splicing assays using the mini‐genes described in (A). Transcripts DNAJC5 (WT), DNAJC5 dUG, and DNAJC5 dC were assessed using qPCR. Results were normalized to snRNA U6 and the fold change value was calculated as described in the methods. Three independent samples were compared for each transcript. Statistics were performed with a one‐way ANOVA and Tukey's post hoc. ***p < 0.001.

Association of TDP43 and hnRNP K and the effects observed on ALS‐related transcripts indicated both could affect alternative splicing regulation. To understand how these two proteins can cooperate during DNAJC5 splicing, we performed an in vitro splicing assay. Given that transcription or NMD degradation does not occur in the in vitro system, this assay could indicate the direct effect of hnRNP K on DNAJC5 splicing. DNAJC5 has unique specific binding sites for TDP43 and hnRNP K on the intron near the cryptical exon region (Figure 6A). Therefore, a set of mini‐genes using a 735 bp sequence spanning the genomic region of DNAJC5 comprising the cryptic exon region between exons 4 and 5 was constructed. The intron in this sequence bears an hnRNP K binding motif, composed by an enriched polyC sequence (poly‐C), and a TDP43 binding motif, formed by an enriched UG sequence (poly‐UG) 8 bp downstream the former (Figure 6A). We performed site‐directed mutagenesis to delete the TDP43 binding motif (DNAJC5 dUG), or the hnRNP K binding motif (DNAJC5 dC) from DNAJC5 wild‐type mini‐gene, generating three different sequences (Figure 6A). After in vitro transcription, we performed in vitro splicing reactions using HeLa nuclear extracts, followed by qPCR to analyze generation of DNAJC5 canonical and cryptic isoforms (Figure 6B). In vitro reactions using DNAJC5 dUG pre‐mRNA, which does not contain the TDP43 binding site, resulted in a significant reduction of DNAJC5cpt isoform (Figure 6B, right panel). We expected an increase of this isoform, since NMD degradation cannot act in vitro. It is possible that other proteins affected splicing of this intron, blocking inclusion of the cryptic exon. In silico analysis performed with the DNAJC5 genomic region comprising exons 4 and 5 revealed other seven RNA‐binding proteins that could affect processing of this intron, including hnRNP K (Figure 6A). However, further experiments are required to confirm that. We also observed a non‐significant reduction on the generation of DNAJC5 canonical isoform (Figure 6B left panel), which corroborates our hypothesis and the results observed with TDP43 KD cells (Figure 1), indicating TDP43 is required for constitutive splicing of DNAJC5.

Absence of hnRNP K binding site on DNAJC5 pre‐mRNA (DNAJC5 dC) did not impact the generation of canonical DNAJC5, since levels observed by RT‐qPCR are similar to those observed on WT transcripts (Figure 6B left panel), reinforcing that hnRNP K possibly acts primarily on transcription and is an auxiliary factor for splicing. DNAJC5 dC transcript cannot associate with hnRNP K, but TDP43 binding sites are intact, and, consistently with TDP43 binding, canonical DNAJC5 levels are normal. However, significantly reduced levels of DNAJC5cpt isoform were observed (Figure 6B right panel). This is consistent with the results observed on hnRNP K KD (see Figure 5), suggesting that hnRNP K is also required for splicing, and for including the cryptic exon in DNAJC5. Altogether, our results indicate that hnRNP K acts during transcription and splicing of DNAJC5. Presence of hnRNP K and TDP43 is important for cryptic exon inclusion and generation of DNAJC5cpt. We reasoned that the interaction of TDP43 and hnRNP K is important for equilibrating DNAJC5 splicing isoforms proportion, where TDP43 is important to promote the canonical DNAJC5 isoform, and hnRNP K is essential for the cryptic isoform production.

From these results, we interpret that TDP43 and hnRNP K activities are strongly connected with the ALS phenotype (A.‐L. Brown et al. 2022; Koike et al. 2023; Moujalled et al. 2017). Upon reduction or removal of TDP43 from the nucleus, preventing transcript binding, there is a reduction on DNAJC5 canonical isoform generation, affecting the proportion of these two isoforms. Even with overexpression of hnRNP K, which stimulates transcription of DNAJC5 pre‐mRNA, under absence of TDP43 it would not be possible to produce sufficient canonical isoform.

### 3.5 Overexpression of hnRNP K Can Affect Endosomal Traffic

**FIGURE 7:**
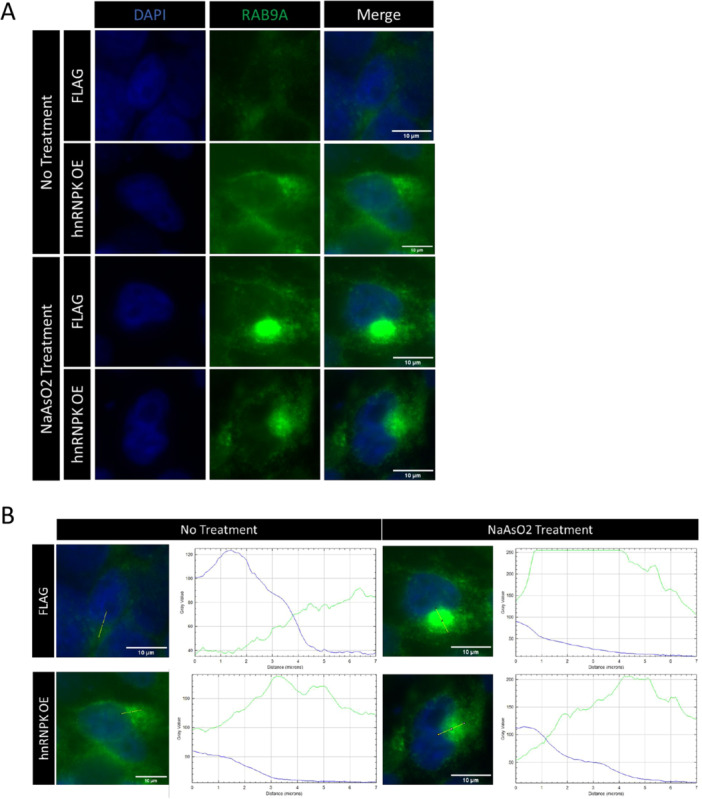
Overexpression of hnRNP K (hnRNP K OE) alters endosomal trafficking. (A) Three independent samples from the hnRNP K OE, FLAG, and sodium arsenite‐treated cells were compared. Cells were transfected with hnRNP K overexpression plasmid (hnRNP K OE) or control plasmid (FLAG) were treated or not with 0.5 mM sodium arsenite for 1 h. Cells were stained with anti‐RAB9A, an endosome marker, conjugated with Alexa Fluor 488 (green). DAPI was used as a nuclear marker (blue). Merge shows both signals together. (B) Representative plot profiles of each FLAG, hnRNP K OE with and without sodium arsenite treatment are shown. A 7 µm‐line was drawn in each image, and the plot profile of blue and green fluorescent intensities (gray value) was captured for the four groups. Scale bars represent 10 µm.

Given that hnRNP K OE can stimulate canonical transcript production of DNAJC5 (Figure 5), and hnRNP K KD reduces canonical levels of DNAJC5 (Figure 6), we wondered whether hnRNP K levels could affect DNAJC5 function. DNAJC5 has been described to be essential for secretion of cytosolic misfolded proteins, acting together with USP19 (Xu et al. 2018). In the MAPS pathway, during proteasome impairment, misfolded proteins attach into USP19 receptors and form endosomes, which carry the cargo outside the cell via late endosomes (Volkmar et al. 2016). Endosomes and late endosomes can be stained with RAB9A antibody (Ganley et al. 2004). We observed hnRNP K OE cells show more RAB9 vesicles than FLAG cells without sodium arsenite treatment (Figure 7A). Upon sodium arsenite treatment of FLAG cells and hnRNP K OE cells, thus mimicking oxidative stress, RAB9A was concentrated and accumulated near the nuclear region, probably the endoplasmic reticulum (Figure 7A and Supplementary Figure 4A). Indeed, hnRNP K OE cells show less concentrated RAB9 vesicles, possibly indicating that hnRNP K facilitates secretion of endosomes due to increased canonical DNAJC5 (Figure 7A and Supplementary Figure 6A). However, comparison of hnRNP K OE and FLAG cells treated with sodium arsenite revealed hnRNP K OE reduced RAB9A accumulation, and RAB9 endosomes seemed more dispersed than the observed in control cells (Figure 7B and Supplementary Figure 6B), suggesting that hnRNP K facilitated endosomal traffic even under oxidative stress, possibly due to regulation of DNAJC5 isoforms.

## 4 Discussion

Alternative splicing is an important mechanism for ALS progression. Many splicing regulators, such as TDP43, FUS, and hnRNP A1, exit the nucleus upon chronic oxidative stress and promote stress granules formation (Ratti et al. 2020; Sidibé et al. 2021). Loss of nuclear TDP43 impairs pre‐mRNA splicing (Rossi and Cozzolino 2021; Seddighi et al. 2024) and promotes alternative transcripts generation, which might lead to disease progression. We hypothesized other protein partners would be important to control TDP43 participation on alternative splicing regulation. In this work, we addressed TDP43 participation on alternative splicing of ALS‐related genes, including *DNAJC5*, which codes for a protein involved with the protein folding pathway (Fontaine et al. 2016; Proenca et al. 2013). We also investigated TDP43 association with the RNA‐binding protein hnRNP K and their cooperation during alternative splicing regulation. Our results indicate these proteins act together on alternative splicing regulation of DNAJC5 during progression of ALS.

ALS progresses upon generation of cryptic isoforms of ATG4B and DNAJC5 (Buratti 2001; Schmidt et al. 2019; Torres et al. 2018). We expected that knockdown of TDP43 would simulate the ALS phenotype, leading to an increase of alternative isoforms. Previous studies indicated absence of TDP43 led to inclusion of cryptic exons in mature mRNAs (Ling et al. 2015), inducing a general reduction on translation of functional proteins (Briese et al. 2020). We observed knockdown of TDP43 resulted in significant reduction of DNAJC5 canonical transcript. This leads to an imbalance of both isoforms, possibly resulting in increased relative levels of the cryptic isoform. NMD inhibition assays performed in TDP43‐KD cells confirmed DNAJC5 cryptic isoforms are degraded, therefore indicating that DNAJC5cpt isoforms are produced upon TDP43 absence.

Overexpression of wild‐type TDP43 did not result in significant changes but mutated versions of TDP43 led to an increase in canonical transcripts. A great part of TDP43 listed mutations have been described in its C‐terminal region (Arseni et al. 2022; Buratti 2015). This region contains two “prion‐like disordered C‐terminal domains” with glutamine/asparagine‐rich regions (Q/N) and glycine‐rich regions, which are known to have an intrinsic disordered region (IDR) (Bolognesi et al. 2019; Fuentealba et al. 2010; Nonaka et al. 2013) and control TDP43 aggregation. Previous studies have shown that missense TDP43^Q331K^ mutation can increase lysine acetylation of TDP43, enhancing its cleavage in the cytoplasm and generating fragments capable of aggregating and increasing cytotoxicity (Kitamura et al. 2016). In addition, cytoplasmic TDP43^Q331K^ can also affect translocation of nuclear proteins as XRCC4, essential for DNA damage signalization, contributing to neurodegeneration (Guerrero et al. 2019). TDP43^Q331K^ and TDP43^M337V^ mutants can also change TDP43 distribution within the cell, from granular‐like disposition to amyloid fibrils‐like, as a consequence of increased physical LLPS aggregation properties, which aggravates the cytotoxicity (Johnson et al. 2009; Lim et al. 2016). Increased TDP43 aggregation also increases the number and size of the stress granules (Lim et al. 2016) and reduces nuclear TDP43 availability. However, our results indicated upregulation of DNAJC5 canonical transcript upon overexpression of TDP43^Q331K^. Overexpression of TDP43^Q331K^ has been described to enhance splicing in specific genes, for example, cryptic exon 17b exclusion in *Sort1*, or exon 2 and 3 inclusion of *MAPT* gene (White et al. 2018). In addition, the increased generation of canonical transcripts upon expression of TDP43^Q331K^ could indicate it has enhanced ability to interact with other partners. On the contrary, for other mutations of TDP43 near residue 331 region, interactomes indicate reduced interactions with SR splicing factors, such as SRSF7, SRSF4, and SF3B3 (Feneberg et al. 2020). The identification of TDP43 partners could help to explain how the splicing machinery will be guided in each gene to induce alternative splicing events.

Previous studies showed TDP43 interacts with other RNA‐binding proteins in specific contexts, specially hnRNP proteins (Freibaum et al. 2010; Geuens et al. 2016). TDP43 can interact with hnRNP U and increase cryptic exon inclusion on POLPID3 transcript (Suzuki et al. 2015). Another work showed that hnRNP A1, hnRNP A2/B1, and hnRNP L can impair cryptic exon inclusion of UNC13A (Koike et al. 2023). Our immunoprecipitation assays confirmed TDP43 associated with hnRNP K in an RNA‐dependent manner within the nuclear region. Previous studies reported that TDP43 is a regulator of hnRNP K expression (Hasegawa‐Ogawa and Okano 2021), and cells bearing TDP43 mutations (TDP43^M337V^ or TDP43^Q331K^) show reduced levels of hnRNP K or altered function, blocking the translation of antioxidant proteins (Moujalled et al. 2017). In addition, recent work described that an alternative isoform of TDP43, without part of its C‐terminal domain, is induced by overexpression of hnRNP K (Hasegawa‐Ogawa et al. 2025). At the time of our experiment, we did not have access of this information and could not check the levels of this alternative isoform, however, we can observe an RNA‐dependent binding interaction between hnRNP K and full‐length TDP43 by our immunoprecipitation assays. Additionally, in vitro splicing data support that both proteins are important for DNAJC5 canonical splicing. We thus interpret that, despite production of TDP43 alternative isoform, full‐length TDP43 was still available upon hnRNP K expression for splicing function.

hnRNP K has been described as a transcription regulator controlling *c‐myc* in cancer (Michelotti et al. 1996). However, it was first reported on alternative splicing, where it has been described as a repressor, affecting splicing of the gene *RunX1*, and the neuronal differentiation of motor neurons in frontotemporal dementia (FTD) (Cao et al. 2012). hnRNP K was also reported to be a repressor of cryptic exon inclusion in transcripts HMBOX 1, TMEM132A, CACTIN1, B4GALNT4, and FAM160B2 (Bampton et al. 2021). Both TDP43 and hnRNP K are involved in transcription and alternative splicing, enhancing or repressing specific profiles (Seguin et al. 2014; Soo et al. 2015). Our data revealed that hnRNP K overexpression increased canonical levels of DNAJC5 and ATG4B transcripts. Accordingly, knockdown of hnRNP K led to reduced levels of both canonical and cryptic DNAJC5 transcripts. Furthermore, our RNA immunoprecipitation assays indicated hnRNP K immunoprecipitates TDP43 and the transcript DNAJC5, suggesting these two proteins are important for cryptic exon splicing of DNAJC5 transcript (Figure 6C). Taken together, our data suggest that TDP43 is essential for splicing of canonical DNAJC5. hnRNP K, on the other hand, is important for transcription control of DNAJC5, since altered expression affects both isoforms tested. In addition, this protein is also important for splicing regulation, stimulating cryptic isoform production. In a non‐pathological situation, it is possible that hnRNP K is controlling the amount of DNAJC5 product, using the splicing mechanism as a negative feedback point. Upon hnRNP K overexpression, canonical isoforms show increased levels. In the ALS condition, despite the effect on transcription, the absence of nuclear TDP43 would promote cryptic exon inclusion of DNAJC5. Given that cryptic isoforms are degraded by NMD, this results in reduced levels of DNAJC5, affecting its function.

DNAJC5 codes for a protein responsible for regulating misfolded protein levels. As part of a family of proteins related to neuronal diseases, other DNAJC proteins are also important for amyotrophic lateral sclerosis development. The gene DNAJC7, which codes for HSP40 (Farhan et al. 2019), is highly expressed during ALS. HSP40 is a chaperone which, together with HSP70, helps to eliminate misfolded proteins (Fan et al. 2003). DNAJC5 was also associated with the MAPS pathway, a non‐canonical mechanism that allows the export of selective cytosolic misfolded proteins (Xu et al. 2018), therefore maintaining the homeostasis in stress conditions. Overexpression of DNAJC5 in neurons together with HSP70, can release Tau protein, associated with Parkinson's disease, from the cells by MAPS pathway (Fontaine et al. 2016). Our immunofluorescence analysis showed that increased levels of hnRNP K can enhance endosomal trafficking, in particular during oxidative stress, probably due to DNAJC5 upregulation. This result strongly indicates that hnRNP K association to TDP43 is important to equilibrate the generation of the alternative isoforms of DNAJC5. In the presence of TDP43 and hnRNP K proteins, production of DNAJC5 is facilitated. Loss of TDP43, as observed in most cases of ALS development and progression, leads to reduced canonical isoforms, as also observed with hnRNP K knockdown. Once in the cytoplasm, a great part of TDP43 concentrates in cytoplasmic stress granules, worsening ALS prognosis (Bampton et al. 2021). It is possible that removal of nuclear TDP43 but retention of hnRNP K during ALS drives to a pathological alternative splicing profile, leading to disease progression. Finally, alterations in DNAJC5 splicing might be essential for defining the prognosis of ALS, since the protein generated is important for protein folding maintenance and misfolded protein vesicle secretion.

In conclusion, our work revealed TDP43 association with hnRNP K is essential for regulating alternative splicing of genes involved in the autophagic and misfolded proteins pathway. Our results indicate that both TDP43 and hnRNP K interact in an RNA‐dependent manner in the nucleus, and hnRNP K affects the regulatory capacity of TDP43 during splicing. In addition, our work suggests the importance of hnRNP K to promote generation of DNAJC5 and maintain vesicle secretion even during oxidative stress.

## Author Contributions

Helder Y. Nagasse and Patricia P. Coltri conceived and designed the research. Helder Y. Nagasse performed the research and acquired the data. Helder Y. Nagasse, Ellen K. Okuda, and Patricia P. Coltri analyzed and interpreted the data. All authors were involved in drafting and revising the manuscript.

## Ethics Statement

This study did not involve experiments with human participants or animals. All experiments were performed using commercially available human cell lines (HEK‐293FT and HeLa) obtained from authenticated repositories and maintained in accordance with institutional biosafety and research guidelines.

## Conflicts of Interest

The authors declare no conflicts of interest.

## Supporting information

Supporting File 1

Supporting File 2

Supporting File 3

Supporting File 4

Supporting File 5

Supporting File 6

## Data Availability

Figures and data are included in the article and supplementary data. Raw data are included in supplementary data. Additional data is available from authors upon reasonable request.

## References

[cbin70158-bib-0001] Arnold, E. S. , S.‐C. Ling , and S. C. Huelga , et al. 2013. “ALS‐Linked TDP‐43 Mutations Produce Aberrant RNA Splicing and Adult‐Onset Motor Neuron Disease Without Aggregation or Loss of Nuclear TDP‐43.” Proceedings of the National Academy of Sciences 110, no. 8: E736–E745. 10.1073/pnas.1222809110. PMC3581922 23382207

[cbin70158-bib-0002] Arseni, D. , M. Hasegawa , A. G. Murzin , et al. 2022. “Structure of Pathological TDP‐43 Filaments From ALS With FTLD.” Nature 601, no. 7891: 139–143. 10.1038/s41586-021-04199-3. 34880495 PMC7612255

[cbin70158-bib-0003] Ayala, Y. M. , L. De Conti , S. E. Avendaño‐Vázquez , et al. 2011. “TDP‐43 Regulates Its mRNA Levels Through a Negative Feedback Loop.” EMBO Journal 30, no. 2: 277–288. 10.1038/emboj.2010.310. 21131904 PMC3025456

[cbin70158-bib-0004] Ayala, Y. M. , P. Zago , A. D'Ambrogio , et al. 2008. “Structural Determinants of the Cellular Localization and Shuttling of TDP‐43.” Journal of Cell Science 121, no. 22: 3778–3785. 10.1242/jcs.038950. 18957508

[cbin70158-bib-0005] Bampton, A. , A. Gatt , J. Humphrey , et al. 2021. “hnRNP K Mislocalisation Is a Novel Protein Pathology of Frontotemporal Lobar Degeneration and Ageing and Leads to Cryptic Splicing.” Acta Neuropathologica 142, no. 4: 609–627. 10.1007/s00401-021-02340-0. 34274995 PMC8423707

[cbin70158-bib-0006] Bolognesi, B. , A. J. Faure , M. Seuma , J. M. Schmiedel , G. G. Tartaglia , and B. Lehner . 2019. “The Mutational Landscape of a Prion‐Like Domain.” Nature Communications 10, no. 1: 4162. 10.1038/s41467-019-12101-z. PMC6744496 31519910

[cbin70158-bib-0007] Bonnal, S. C. , I. López‐Oreja , and J. Valcárcel . 2020. “Roles and Mechanisms of Alternative Splicing in Cancer — Implications for Care.” Nature Reviews Clinical Oncology 17, no. 8: 457–474. 10.1038/s41571-020-0350-x. 32303702

[cbin70158-bib-0008] Brandão‐Teles, C. , A. S. L. M. Antunes , T. A. de Moraes Vrechi , and D. Martins‐de‐Souza . 2024. “The Roles of hnRNP Family in the Brain and Brain‐Related Disorders.” Molecular Neurobiology 61, no. 6: 3578–3595. 10.1007/s12035-023-03747-4. 37999871

[cbin70158-bib-0009] Briese, M. , L. Saal‐Bauernschubert , P. Lüningschrör , et al. 2020. “Loss of Tdp‐43 Disrupts the Axonal Transcriptome of Motoneurons Accompanied by Impaired Axonal Translation and Mitochondria Function.” Acta Neuropathologica Communications 8, no. 1: 116. 10.1186/s40478-020-00987-6. 32709255 PMC7379803

[cbin70158-bib-0010] Brown, A.‐L. , O. G. Wilkins , M. J. Keuss , et al. 2022. “TDP‐43 Loss and ALS‐Risk SNPs Drive Mis‐Splicing and Depletion of UNC13A.” Nature 603, no. 7899: 131–137. 10.1038/s41586-022-04436-3. 35197628 PMC8891020

[cbin70158-bib-0011] Brown, R. H. , and A. Al‐Chalabi . 2017. “Amyotrophic Lateral Sclerosis.” New England Journal of Medicine 377, no. 2: 162–172. 10.1056/NEJMra1603471. 28700839

[cbin70158-bib-0012] Buratti, E. 2001. “Nuclear Factor TDP‐43 and SR Proteins Promote In Vitro and In Vivo CFTR Exon 9 Skipping.” EMBO Journal 20, no. 7: 1774–1784. 10.1093/emboj/20.7.1774. 11285240 PMC145463

[cbin70158-bib-0013] Buratti, E. 2015. “Functional Significance of TDP‐43 Mutations in Disease.” Advances in Genetics 91: 1–53. 10.1016/bs.adgen.2015.07.001. 26410029

[cbin70158-bib-0014] Cairns, N. J. , M. Neumann , E. H. Bigio , et al. 2007. “TDP‐43 in Familial and Sporadic Frontotemporal Lobar Degeneration With Ubiquitin Inclusions.” American Journal of Pathology 171, no. 1: 227–240. 10.2353/ajpath.2007.070182. 17591968 PMC1941578

[cbin70158-bib-0015] Cao, W. , A. Razanau , D. Feng , V. G. Lobo , and J. Xie . 2012. “Control of Alternative Splicing by Forskolin Through hnRNP K During Neuronal Differentiation.” Nucleic Acids Research 40, no. 16: 8059–8071. 10.1093/nar/gks504. 22684629 PMC3439897

[cbin70158-bib-0016] Dejgaard, K. , H. Leffers , H. H. Rasmussen , et al. 1994. “Identification, Molecular Cloning, Expression and Chromosome Mapping of a Family of Transformation Upregulated hnRNP‐K Proteins Derived by Alternative Splicing.” Journal of Molecular Biology 236, no. 1: 33–48. 10.1006/jmbi.1994.1116. 8107114

[cbin70158-bib-0017] Ding, M. , D. Wang , H. Chen , et al. 2025. “A Biophysical Basis for the Spreading Behavior and Limited Diffusion of Xist.” Cell 188, no. 4: 978–997.e25. 10.1016/j.cell.2024.12.004. 39824183 PMC11863002

[cbin70158-bib-0018] Dreyfuss, G. , M. J. Matunis , S. Pinol‐Roma , and C. G. Burd . 1993. “hnRNP Proteins and the Biogenesis of mRNA.” Annual Review of Biochemistry 62, no. 1: 289–321. 10.1146/annurev.bi.62.070193.001445. 8352591

[cbin70158-bib-0019] Etoc, F. , J. Metzger , A. Ruzo , et al. 2016. “A Balance Between Secreted Inhibitors and Edge Sensing Controls Gastruloid Self‐Organization.” Developmental Cell 39, no. 3: 302–315. 10.1016/j.devcel.2016.09.016. 27746044 PMC5113147

[cbin70158-bib-0020] Fan, C.‐Y. , S. Lee , and D. M. Cyr . 2003. “Mechanisms for Regulation of Hsp70 Function by Hsp40.” Cell Stress & Chaperones 8, no. 4: 309–316. 10.1379/1466-1268(2003)008<0309:MFROHF>2.0.CO;2. 15115283 PMC514902

[cbin70158-bib-0021] Farhan, S. M. K. , D. P. Howrigan , L. E. Abbott , et al. 2019. “Exome Sequencing in Amyotrophic Lateral Sclerosis Implicates a Novel Gene, DNAJC7, Encoding a Heat‐Shock Protein.” Nature Neuroscience 22, no. 12: 1966–1974. 10.1038/s41593-019-0530-0. 31768050 PMC6919277

[cbin70158-bib-0022] Feneberg, E. , D. Gordon , A. G. Thompson , et al. 2020. “An ALS‐Linked Mutation in TDP‐43 Disrupts Normal Protein Interactions in the Motor Neuron Response to Oxidative Stress.” Neurobiology of Disease 144: 105050. 10.1016/j.nbd.2020.105050. 32800996

[cbin70158-bib-0023] Fontaine, S. N. , D. Zheng , J. J. Sabbagh , et al. 2016. “DnaJ/Hsc70 Chaperone Complexes Control the Extracellular Release of Neurodegenerative‐Associated Proteins.” EMBO Journal 35, no. 14: 1537–1549. 10.15252/embj.201593489. 27261198 PMC4946142

[cbin70158-bib-0024] Fratta, P. , P. Sivakumar , and J. Humphrey , et al. 2018. “Mice With Endogenous TDP‐43 Mutations Exhibit Gain of Splicing Function and Characteristics of Amyotrophic Lateral Sclerosis.” EMBO Journal 37, no. 11: EMBJ201798684. 10.15252/embj.201798684. PMC5983119 29764981

[cbin70158-bib-0025] Freibaum, B. D. , R. K. Chitta , A. A. High , and J. P. Taylor . 2010. “Global Analysis of TDP‐43 Interacting Proteins Reveals Strong Association With RNA Splicing and Translation Machinery.” Journal of Proteome Research 9, no. 2: 1104–1120. 10.1021/pr901076y. 20020773 PMC2897173

[cbin70158-bib-0026] Fuentealba, R. A. , M. Udan , S. Bell , et al. 2010. “Interaction With Polyglutamine Aggregates Reveals a Q/N‐Rich Domain in TDP‐43.” Journal of Biological Chemistry 285, no. 34: 26304–26314. 10.1074/jbc.M110.125039. 20554523 PMC2924052

[cbin70158-bib-0027] Ganley, I. G. , K. Carroll , L. Bittova , and S. Pfeffer . 2004. “Rab9 GTPase Regulates Late Endosome Size and Requires Effector Interaction for Its Stability.” Molecular Biology of the Cell 15, no. 12: 5420–5430. 10.1091/mbc.e04-08-0747. 15456905 PMC532021

[cbin70158-bib-0028] Gasset‐Rosa, F. , S. Lu , H. Yu , et al. 2019. “Cytoplasmic TDP‐43 De‐Mixing Independent of Stress Granules Drives Inhibition of Nuclear Import, Loss of Nuclear TDP‐43, and Cell Death.” Neuron 102, no. 2: 339–357.e7. 10.1016/j.neuron.2019.02.038. 30853299 PMC6548321

[cbin70158-bib-0029] Geuens, T. , D. Bouhy , and V. Timmerman . 2016. “The hnRNP Family: Insights Into Their Role in Health and Disease.” Human Genetics 135, no. 8: 851–867. 10.1007/s00439-016-1683-5. 27215579 PMC4947485

[cbin70158-bib-0030] Grad, L. I. , G. A. Rouleau , J. Ravits , and N. R. Cashman . 2017. “Clinical Spectrum of Amyotrophic Lateral Sclerosis (ALS).” Cold Spring Harbor Perspectives in Medicine 7, no. 8: a024117. 10.1101/cshperspect.a024117. 28003278 PMC5538408

[cbin70158-bib-0031] Green, M. , and J. Sambrook . 2012. Molecular Cloning: A Laboratory Manual (2, 4th Edition. Cold Spring Harbor Laboratory Press.

[cbin70158-bib-0032] Guarracino, A. , G. Pepe , F. Ballesio , et al. 2021. “BRIO: A Web Server for RNA Sequence and Structure Motif Scan.” Nucleic Acids Research 49, no. W1: W67–W71. 10.1093/nar/gkab400. 34038531 PMC8262756

[cbin70158-bib-0033] Guerrero, E. N. , J. Mitra , H. Wang , et al. 2019. “Amyotrophic Lateral Sclerosis‐Associated TDP‐43 Mutation Q331K Prevents Nuclear Translocation of XRCC4‐DNA Ligase 4 Complex and Is Linked to Genome Damage‐Mediated Neuronal Apoptosis.” Human Molecular Genetics 28, no. 15: 2459–2476. 10.1093/hmg/ddz062. 31067307 PMC6659010

[cbin70158-bib-0034] Hallegger, M. , A. M. Chakrabarti , F. C. Y. Lee , et al. 2021. “TDP‐43 Condensation Properties Specify Its RNA‐Binding and Regulatory Repertoire.” Cell 184, no. 18: 4680–4696.e22. 10.1016/j.cell.2021.07.018. 34380047 PMC8445024

[cbin70158-bib-0035] Hasegawa‐Ogawa, M. , and H. J. Okano . 2021. “Characterization of the Upstream and Intron Promoters of the Gene Encoding TAR DNA‐Binding Protein.” Scientific Reports 11, no. 1: 8720. 10.1038/s41598-021-88015-y. 33888768 PMC8062691

[cbin70158-bib-0036] Hasegawa‐Ogawa, M. , A. Onda‐Ohto , and T. Nakajo , et al. 2025. “Dominant‐Negative Isoform of TDP‐43 Is Regulated by ALS‐Linked RNA‐Binding Proteins.” Journal of Cell Biology 224, no. 10: e202406097. 10.1083/jcb.202406097. 40778857 PMC12333503

[cbin70158-bib-0037] Ishigaki, Y. , X. Li , G. Serin , and L. E. Maquat . 2001. “Evidence for a Pioneer Round of mRNA Translation.” Cell 106, no. 5: 607–617. 10.1016/S0092-8674(01)00475-5. 11551508

[cbin70158-bib-0038] Janssens, J. , H. Wils , G. Kleinberger , et al. 2013. “Overexpression of ALS‐Associated p.M337V Human TDP‐43 in Mice Worsens Disease Features Compared to Wild‐Type Human TDP‐43 Mice.” Molecular Neurobiology 48, no. 1: 22–35. 10.1007/s12035-013-8427-5. 23475610 PMC3718993

[cbin70158-bib-0039] Johnson, B. S. , D. Snead , J. J. Lee , J. M. McCaffery , J. Shorter , and A. D. Gitler . 2009. “TDP‐43 Is Intrinsically Aggregation‐Prone, and Amyotrophic Lateral Sclerosis‐Linked Mutations Accelerate Aggregation and Increase Toxicity.” Journal of Biological Chemistry 284, no. 30: 20329–20339. 10.1074/jbc.M109.010264. 19465477 PMC2740458

[cbin70158-bib-0040] Kapustin, Y. , E. Chan , R. Sarkar , et al. 2011. “Cryptic Splice Sites and Split Genes.” Nucleic Acids Research 39, no. 14: 5837–5844. 10.1093/nar/gkr203. 21470962 PMC3152350

[cbin70158-bib-0041] Kawahara, Y. , and A. Mieda‐Sato . 2012. “TDP‐43 Promotes microRNA Biogenesis as a Component of the Drosha and Dicer Complexes.” Proceedings of the National Academy of Sciences 109, no. 9: 3347–3352. 10.1073/pnas.1112427109. PMC3295278 22323604

[cbin70158-bib-0042] Kitamura, A. , Y. Nakayama , A. Shibasaki , et al. 2016. “Interaction of RNA With a C‐Terminal Fragment of the Amyotrophic Lateral Sclerosis‐Associated TDP43 Reduces Cytotoxicity.” Scientific Reports 6, no. 1: 19230. 10.1038/srep19230. 26757674 PMC4725827

[cbin70158-bib-0043] Klim, J. R. , L. A. Williams , F. Limone , et al. 2019. “ALS‐Implicated Protein TDP‐43 Sustains Levels of STMN2, a Mediator of Motor Neuron Growth and Repair.” Nature Neuroscience 22, no. 2: 167–179. 10.1038/s41593-018-0300-4. 30643292 PMC7153761

[cbin70158-bib-0044] Koike, Y. , S. Pickles , V. Estades Ayuso , et al. 2023. “TDP‐43 and Other hnRNPs Regulate Cryptic Exon Inclusion of a Key ALS/FTD Risk Gene, UNC13A.” PLoS Biology 21, no. 3: e3002028. 10.1371/journal.pbio.3002028. 36930682 PMC10057836

[cbin70158-bib-0045] Krecic, A. M. , and M. S. Swanson . 1999. “hnRNP Complexes: Composition, Structure, and Function.” Current Opinion in Cell Biology 11, no. 3: 363–371. 10.1016/S0955-0674(99)80051-9. 10395553

[cbin70158-bib-0046] Lee, Y. , and D. C. Rio . 2015. “Mechanisms and Regulation of Alternative Pre‐mRNA Splicing.” Annual Review of Biochemistry 84, no. 1: 291–323. 10.1146/annurev-biochem-060614-034316. PMC4526142 25784052

[cbin70158-bib-0047] Li, Y. , H. Wang , J. Wan , Q. Ma , Y. Qi , and Z. Gu . 2022. “The hnRNPK/A1/R/U Complex Regulates Gene Transcription and Translation and Is a Favorable Prognostic Biomarker for Human Colorectal Adenocarcinoma.” Frontiers in Oncology 12: 845931. 10.3389/fonc.2022.845931. 35875075 PMC9301189

[cbin70158-bib-0048] Li, Z. , J. K. Vuong , M. Zhang , C. Stork , and S. Zheng . 2017. “Inhibition of Nonsense‐Mediated RNA Decay by ER Stress.” RNA 23, no. 3: 378–394. 10.1261/rna.058040.116. 27940503 PMC5311500

[cbin70158-bib-0049] Lim, L. , Y. Wei , Y. Lu , and J. Song . 2016. “ALS‐Causing Mutations Significantly Perturb the Self‐Assembly and Interaction With Nucleic Acid of the Intrinsically Disordered Prion‐Like Domain of TDP‐43.” PLoS Biology 14, no. 1: e1002338. 10.1371/journal.pbio.1002338. 26735904 PMC4703307

[cbin70158-bib-0050] Ling, J. P. , O. Pletnikova , J. C. Troncoso , and P. C. Wong . 2015. “TDP‐43 Repression of Nonconserved Cryptic Exons Is Compromised in ALS‐FTD.” Science 349, no. 6248: 650–655. 10.1126/science.aab0983. 26250685 PMC4825810

[cbin70158-bib-0051] Mayeda, A. , and A. R. Krainer . 1992. “Regulation of Alternative Pre‐mRNA Splicing by hnRNP A1 and Splicing Factor SF2.” Cell 68, no. 2: 365–375. 10.1016/0092-8674(92)90477-T. 1531115

[cbin70158-bib-0052] Michelotti, E. F. , G. A. Michelotti , A. I. Aronsohn , and D. Levens . 1996. “Heterogeneous Nuclear Ribonucleoprotein K Is a Transcription Factor.” Molecular and Cellular Biology 16, no. 5: 2350–2360. 10.1128/MCB.16.5.2350. 8628302 PMC231223

[cbin70158-bib-0053] Mohagheghi, F. , M. Prudencio , C. Stuani , et al. 2016. “TDP‐43 Functions Within a Network of hnRNP Proteins to Inhibit the Production of a Truncated Human SORT1 Receptor.” Human Molecular Genetics 25, no. 3: 534–545. 10.1093/hmg/ddv491. 26614389 PMC4731020

[cbin70158-bib-0054] Morera, A. A. , N. S. Ahmed , and J. C. Schwartz . 2019. “TDP‐43 Regulates Transcription at Protein‐Coding Genes and Alu Retrotransposons.” Biochimica et Biophysica Acta (BBA) ‐ Gene Regulatory Mechanisms 1862, no. 10: 194434. 10.1016/j.bbagrm.2019.194434. 31655156 PMC6899060

[cbin70158-bib-0055] Moujalled, D. , A. Grubman , K. Acevedo , et al. 2017. “TDP‐43 Mutations Causing Amyotrophic Lateral Sclerosis Are Associated With Altered Expression of RNA‐Binding Protein hnRNP K and Affect the Nrf2 Antioxidant Pathway.” Human Molecular Genetics 26, no. 9: 1732–1746. 10.1093/hmg/ddx093. 28334913

[cbin70158-bib-0056] Moujalled, D. , J. L. James , S. Yang , et al. 2015. “Phosphorylation of hnRNP K by Cyclin‐Dependent Kinase 2 Controls Cytosolic Accumulation of TDP‐43.” Human Molecular Genetics 24, no. 6: 1655–1669. 10.1093/hmg/ddu578. 25410660

[cbin70158-bib-0057] Neelagandan, N. , G. Gonnella , S. Dang , et al. 2019. “TDP‐43 Enhances Translation of Specific mRNAs Linked to Neurodegenerative Disease.” Nucleic Acids Research 47, no. 1: 341–361. 10.1093/nar/gky972. 30357366 PMC6326785

[cbin70158-bib-0058] Nilsen, T. W. 2013. “Preparation of Nuclear Extracts From HeLa Cells.” Cold Spring Harbor Protocols 2013, no. 6: pdb.prot075176. 10.1101/pdb.prot075176. 23734028

[cbin70158-bib-0059] Nonaka, T. , M. Masuda‐Suzukake , T. Arai , et al. 2013. “Prion‐Like Properties of Pathological TDP‐43 Aggregates From Diseased Brains.” Cell Reports 4, no. 1: 124–134. 10.1016/j.celrep.2013.06.007. 23831027

[cbin70158-bib-0060] Nosková, L. , V. Stránecký , H. Hartmannová , et al. 2011. “Mutations in DNAJC5, Encoding Cysteine‐String Protein Alpha, Cause Autosomal‐Dominant Adult‐Onset Neuronal Ceroid Lipofuscinosis.” American Journal of Human Genetics 89, no. 2: 241–252. 10.1016/j.ajhg.2011.07.003. 21820099 PMC3155175

[cbin70158-bib-0061] Oiwa, K. , S. Watanabe , and K. Onodera , et al. 2023. “Monomerization of TDP‐43 Is a Key Determinant for Inducing TDP‐43 Pathology in Amyotrophic Lateral Sclerosis.” Science Advances 9, no. 31: adf6895. 10.1126/sciadv.adf6895. PMC10403219 37540751

[cbin70158-bib-0062] Panda, S. K. , S. V. Boddul , G. Y. Jiménez‐Andrade , et al. 2017. “Green Listed—A CRISPR Screen Tool.” Bioinformatics 33, no. 7: 1099–1100. 10.1093/bioinformatics/btw739. 28414855

[cbin70158-bib-0063] Polymenidou, M. , C. Lagier‐Tourenne , K. R. Hutt , et al. 2011. “Long Pre‐mRNA Depletion and RNA Missplicing Contribute to Neuronal Vulnerability From Loss of TDP‐43.” Nature Neuroscience 14, no. 4: 459–468. 10.1038/nn.2779. 21358643 PMC3094729

[cbin70158-bib-0064] Prasad, A. , V. Bharathi , V. Sivalingam , A. Girdhar , and B. K. Patel . 2019. “Molecular Mechanisms of TDP‐43 Misfolding and Pathology in Amyotrophic Lateral Sclerosis.” Frontiers in Molecular Neuroscience 12: 25. 10.3389/fnmol.2019.00025. 30837838 PMC6382748

[cbin70158-bib-0065] Proenca, C. C. , N. Stoehr , M. Bernhard , et al. 2013. “Atg4b‐Dependent Autophagic Flux Alleviates Huntington's Disease Progression.” PLoS One 8, no. 7: e68357. 10.1371/journal.pone.0068357. 23861892 PMC3704647

[cbin70158-bib-0066] Purice, M. D. , and J. P. Taylor . 2018. “Linking hnRNP Function to ALS and FTD Pathology.” Frontiers in Neuroscience 12: 326. 10.3389/fnins.2018.00326. 29867335 PMC5962818

[cbin70158-bib-0067] Ratti, A. , V. Gumina , P. Lenzi , et al. 2020. “Chronic Stress Induces Formation of Stress Granules and Pathological TDP‐43 Aggregates in Human ALS Fibroblasts and iPSC‐Motoneurons.” Neurobiology of Disease 145: 105051. 10.1016/j.nbd.2020.105051. 32827688

[cbin70158-bib-0068] Rossi, S. , and M. Cozzolino . 2021. “Dysfunction of RNA/RNA‐Binding Proteins in ALS Astrocytes and Microglia.” Cells 10, no. 11: 3005. 10.3390/cells10113005. 34831228 PMC8616248

[cbin70158-bib-0069] Russell, R. , A. W. Karzai , A. F. Mehl , and R. McMacken . 1999. “DnaJ Dramatically Stimulates ATP Hydrolysis by DnaK: Insight Into Targeting of Hsp70 Proteins to Polypeptide Substrates.” Biochemistry 38, no. 13: 4165–4176. 10.1021/bi9824036. 10194333

[cbin70158-bib-0070] Schmidt, H. B. , A. Barreau , and R. Rohatgi . 2019. “Phase Separation‐Deficient TDP43 Remains Functional in Splicing.” Nature Communications 10, no. 1: 4890. 10.1038/s41467-019-12740-2. PMC6814767 31653829

[cbin70158-bib-0071] Seddighi, S. , Y. A. Qi , and A.‐L. Brown , et al. 2024. “Mis‐Spliced Transcripts Generate De Novo Proteins in TDP‐43–Related ALS/FTD.” Science Translational Medicine 16, no. 734: eadg7162. 10.1126/scitranslmed.adg7162. 38277467 PMC11325748

[cbin70158-bib-0072] Seguin, S. J. , F. F. Morelli , J. Vinet , et al. 2014. “Inhibition of Autophagy, Lysosome and VCP Function Impairs Stress Granule Assembly.” Cell Death and Differentiation 21, no. 12: 1838–1851. 10.1038/cdd.2014.103. 25034784 PMC4227144

[cbin70158-bib-0073] Sidibé, H. , Y. Khalfallah , S. Xiao , et al. 2021. “TDP‐43 Stabilizes *G3BP1* mRNA: Relevance to Amyotrophic Lateral Sclerosis/Frontotemporal Dementia.” Brain 144, no. 11: 3461–3476. 10.1093/brain/awab217. 34115105 PMC8677511

[cbin70158-bib-0074] Soo, K. Y. , M. Halloran , V. Sundaramoorthy , et al. 2015. “Rab1‐Dependent ER–Golgi Transport Dysfunction Is a Common Pathogenic Mechanism in SOD1, TDP‐43 and FUS‐Associated ALS.” Acta Neuropathologica 130, no. 5: 679–697. 10.1007/s00401-015-1468-2. 26298469

[cbin70158-bib-0075] Suzuki, H. , Y. Shibagaki , S. Hattori , and M. Matsuoka . 2015. “Nuclear TDP‐43 Causes Neuronal Toxicity by Escaping From the Inhibitory Regulation by hnRNPs.” Human Molecular Genetics 24, no. 6: 1513–1527. 10.1093/hmg/ddu563. 25378556

[cbin70158-bib-0076] Torres, P. , O. Ramírez‐Núñez , R. Romero‐Guevara , et al. 2018. “Cryptic Exon Splicing Function of TARDBP Interacts With Autophagy in Nervous Tissue.” Autophagy 14, no. 8: 1398–1403. 10.1080/15548627.2018.1474311. 29912613 PMC6103657

[cbin70158-bib-0077] Torres, P. , S. Rico‐Rios , M. Ceron‐Codorniu , et al. 2024. “TDP‐43 Regulates LC3ylation in Neural Tissue Through ATG4B Cryptic Splicing Inhibition.” Acta Neuropathologica 148, no. 1: 45. 10.1007/s00401-024-02780-4. 39305312 PMC11416411

[cbin70158-bib-0078] Valverde, R. , L. Edwards , and L. Regan . 2008. “Structure and Function of KH Domains.” FEBS Journal 275, no. 11: 2712–2726. 10.1111/j.1742-4658.2008.06411.x. 18422648

[cbin70158-bib-0079] Veraldi, K. L. , G. K. Arhin , K. Martincic , L.‐H. Chung‐Ganster , J. Wilusz , and C. Milcarek . 2001. “hnRNP F Influences Binding of a 64‐Kilodalton Subunit of Cleavage Stimulation Factor to mRNA Precursors in Mouse B Cells.” Molecular and Cellular Biology 21, no. 4: 1228–1238. 10.1128/MCB.21.4.1228-1238.2001. 11158309 PMC99576

[cbin70158-bib-0080] Volkmar, N. , E. Fenech , and J. C. Christianson . 2016. “New MAPS for Misfolded Proteins.” Nature Cell Biology 18, no. 7: 724–726. 10.1038/ncb3381. 27350445

[cbin70158-bib-0081] Wheeler, J. R. , T. Matheny , S. Jain , R. Abrisch , and R. Parker . 2016. “Distinct Stages in Stress Granule Assembly and Disassembly.” eLife 5: e18413. 10.7554/eLife.18413. 27602576 PMC5014549

[cbin70158-bib-0082] White, M. A. , E. Kim , A. Duffy , et al. 2018. “TDP‐43 Gains Function Due to Perturbed Autoregulation in a Tardbp Knock‐In Mouse Model of ALS‐FTD.” Nature Neuroscience 21, no. 4: 552–563. 10.1038/s41593-018-0113-5. 29556029 PMC5884423

[cbin70158-bib-0083] Winton, M. J. , L. M. Igaz , M. M. Wong , L. K. Kwong , J. Q. Trojanowski , and V. M.‐Y. Lee . 2008. “Disturbance of Nuclear and Cytoplasmic TAR DNA‐Binding Protein (TDP‐43) Induces Disease‐Like Redistribution, Sequestration, and Aggregate Formation.” Journal of Biological Chemistry 283, no. 19: 13302–13309. 10.1074/jbc.M800342200. 18305110 PMC2442318

[cbin70158-bib-0084] Wolozin, B. , and P. Ivanov . 2019. “Stress Granules and Neurodegeneration.” Nature Reviews Neuroscience 20, no. 11: 649–666. 10.1038/s41583-019-0222-5. 31582840 PMC6986315

[cbin70158-bib-0085] Xu, Y. , L. Cui , A. Dibello , et al. 2018. “DNAJC5 Facilitates USP19‐Dependent Unconventional Secretion of Misfolded Cytosolic Proteins.” Cell Discovery 4, no. 1: 11. 10.1038/s41421-018-0012-7. 29531792 PMC5838229

[cbin70158-bib-0086] Xu, Y. , R. Li , K. Zhang , et al. 2018. “The Multifunctional RNA‐Binding Protein hnRNPK Is Critical for the Proliferation and Differentiation of Myoblasts.” BMB Reports 51, no. 7: 350–355. 10.5483/BMBRep.2018.51.7.043. 29898807 PMC6089871

