# Supplementary material for: TDP43 and hnRNP K Regulate Alternative Splicing of DNAJC5
Source: Cell Biol Int. 2026 Apr 15;50(4):e70158. doi: 10.1002/cbin.70158 (PMC13081507; doi:10.1002/cbin.70158)

Supplementary Figure 1

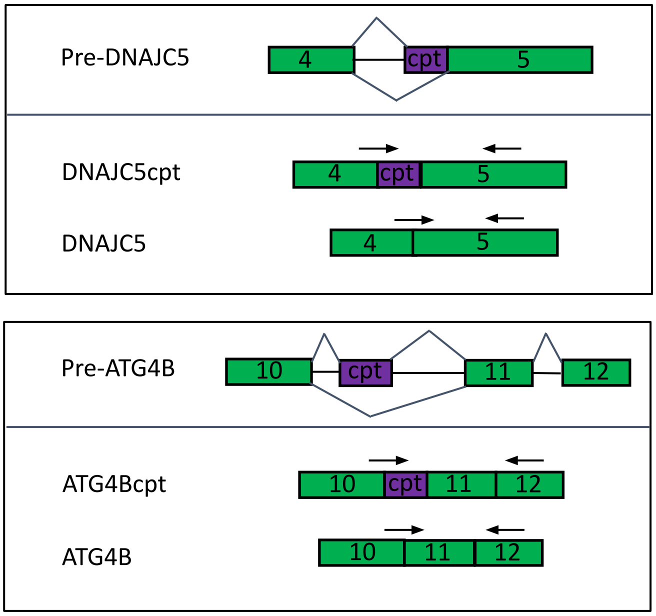

Supplementary Figure 2

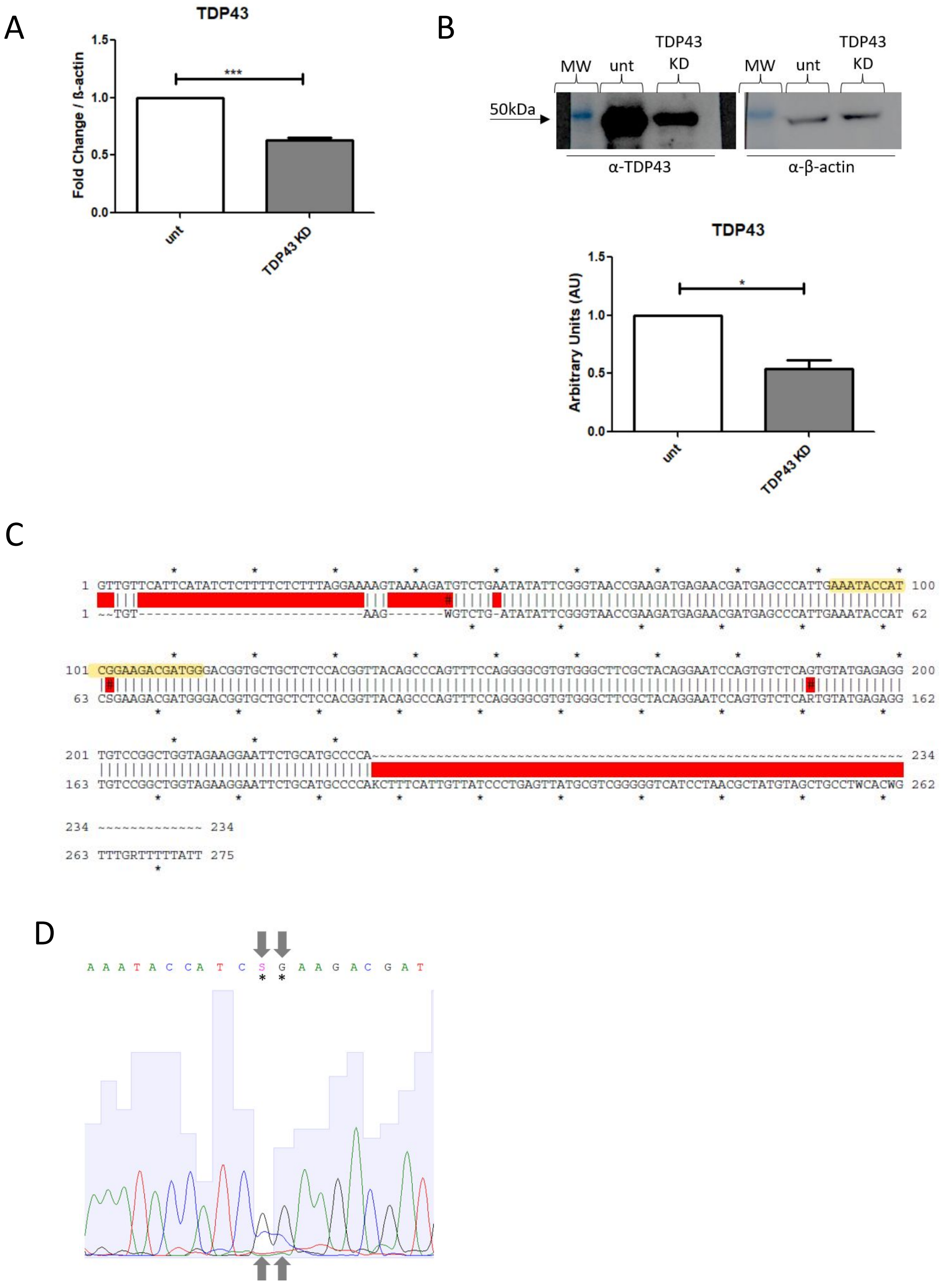

Supplementary Figure 3

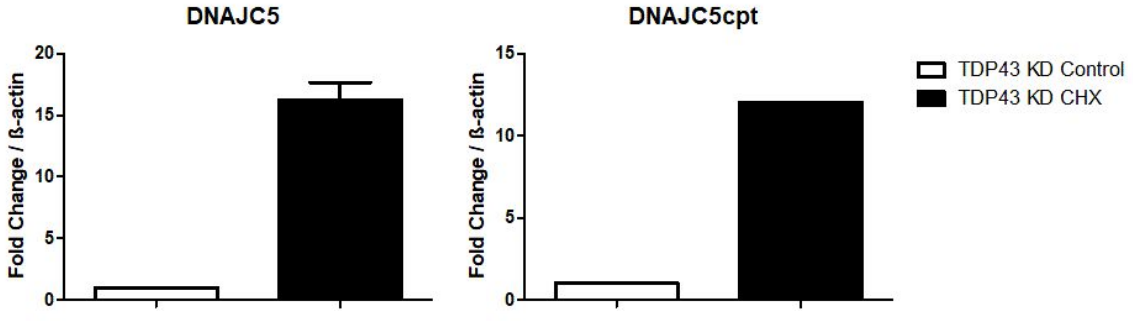

Supplementary Figure 4

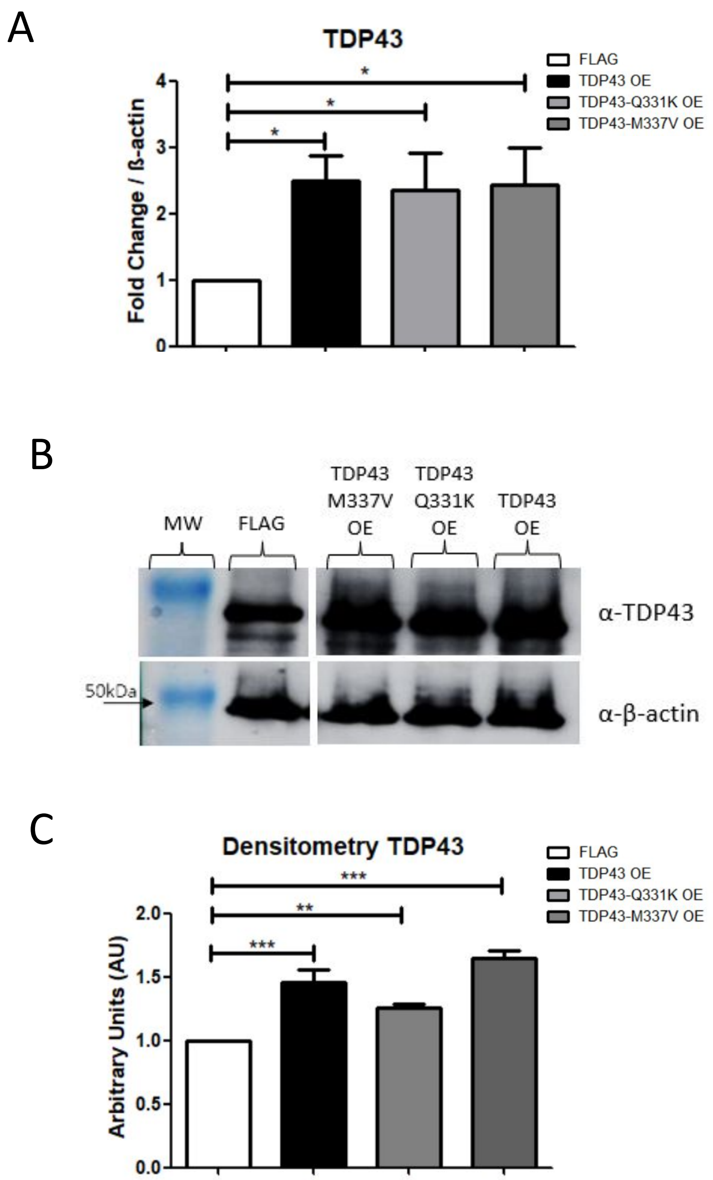

Supplementary Figure 5

A

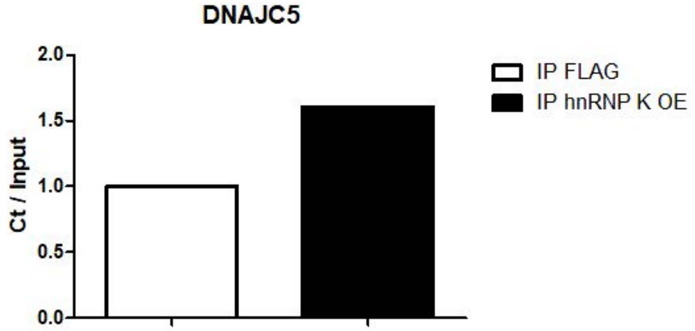

Supplementary Figure 6

A

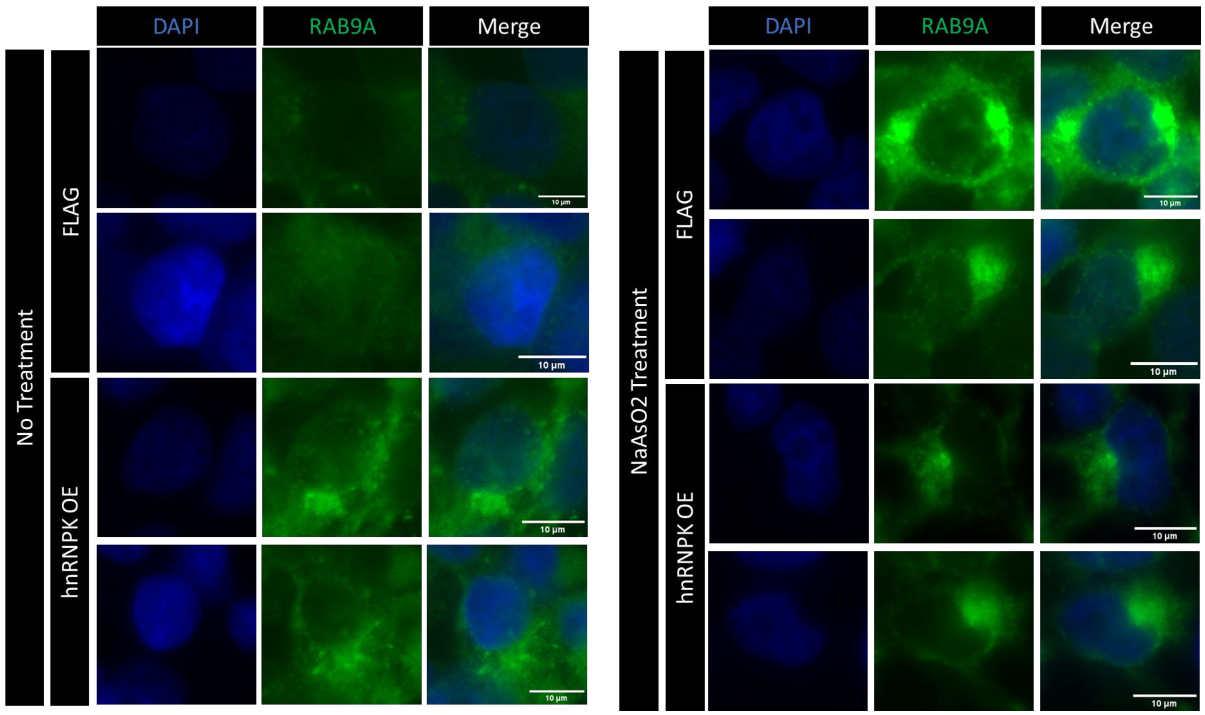

B

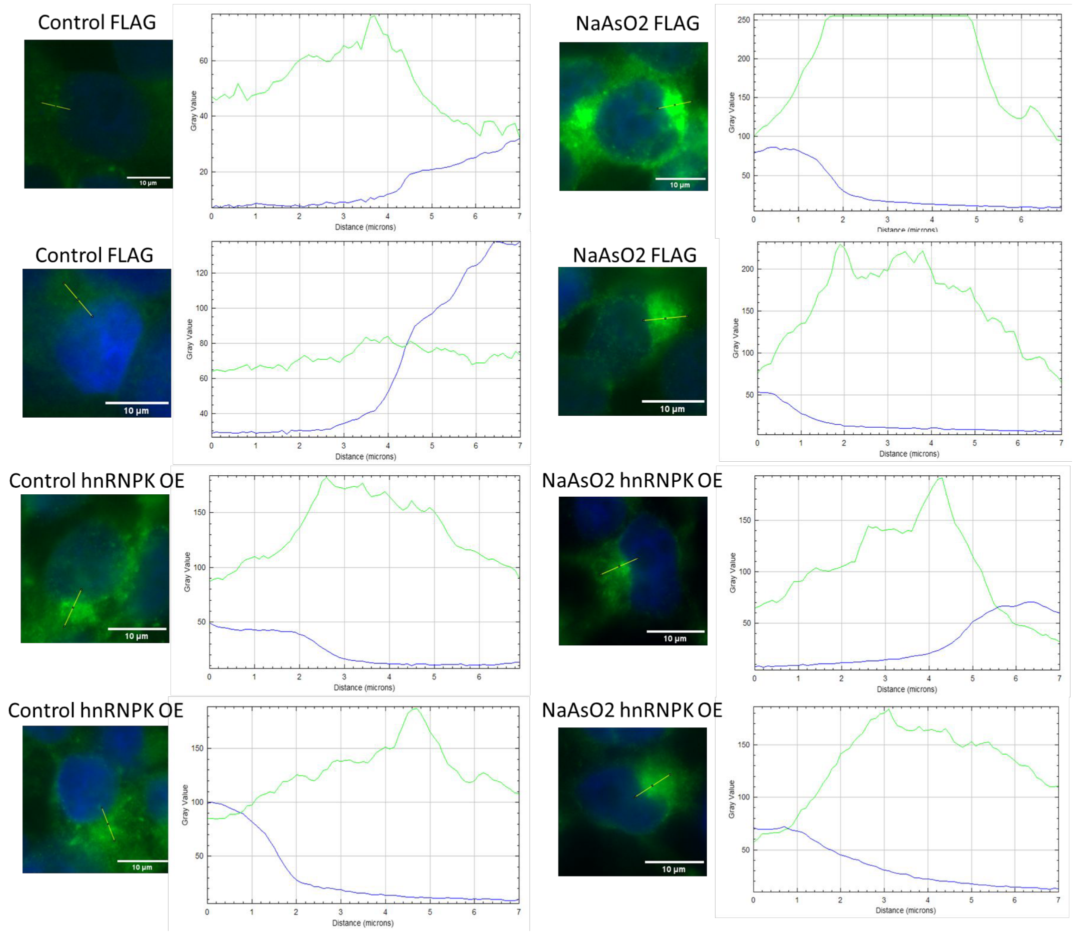

Supplement: Supplementary file 1 — Supporting File 1 [file CBIN-50-0-s006.pdf]
