# Supplementary figures and images for: TDP43 and hnRNP K Regulate Alternative Splicing of DNAJC5
Source: Cell Biol Int. 2026 Apr 15;50(4):e70158. doi: 10.1002/cbin.70158 (PMC13081507; doi:10.1002/cbin.70158)

Supplementary Figure 2

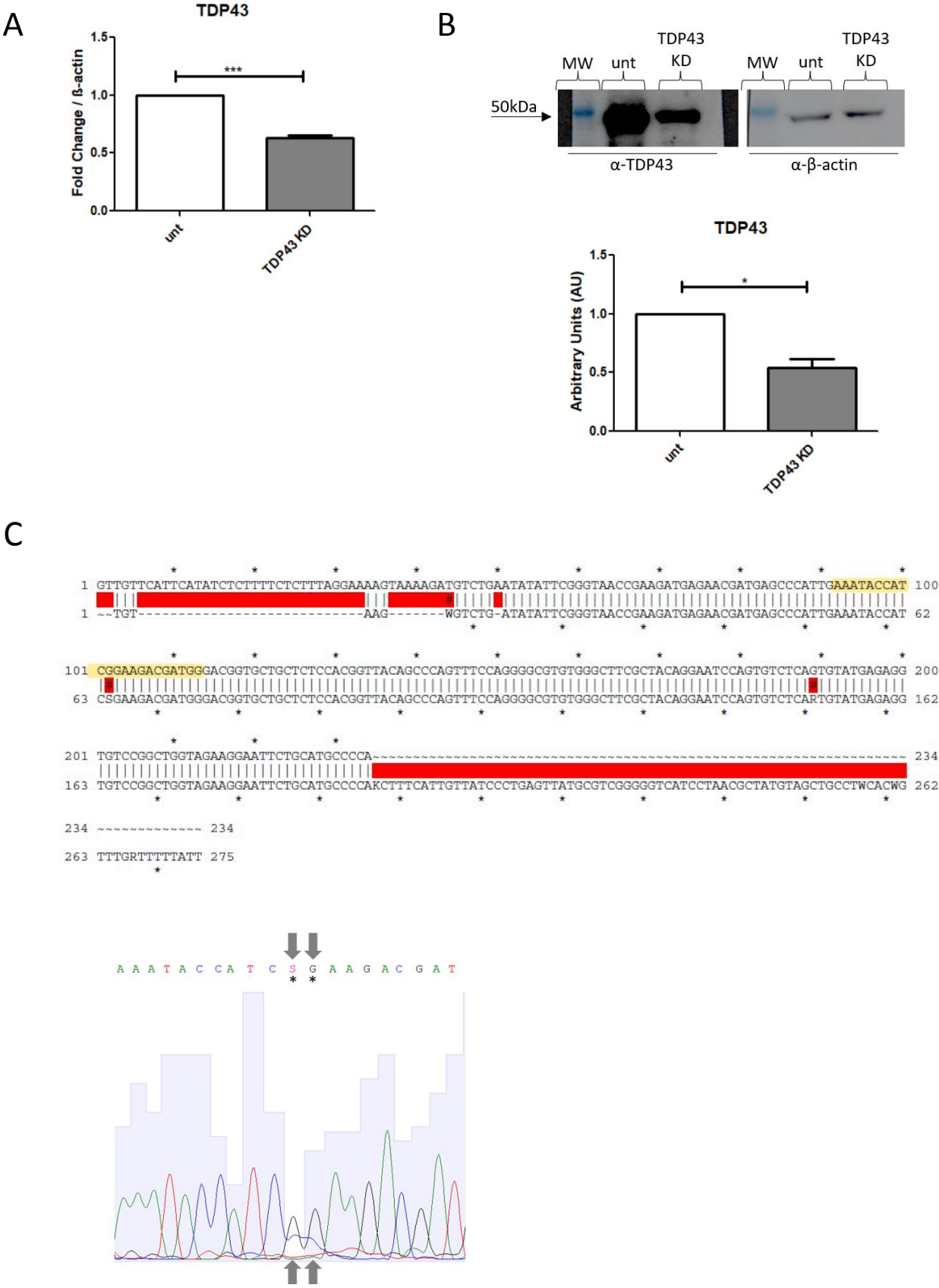

Supplement: Supplementary file 2 — Supporting File 2 [file CBIN-50-0-s004.pdf]

Supplementary Figure 3

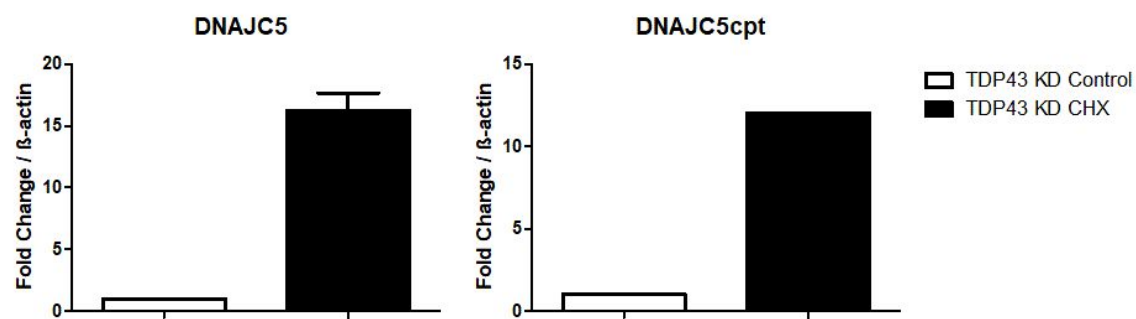

Supplement: Supplementary file 3 — Supporting File 3 [file CBIN-50-0-s002.pdf]

# Supplementary Figure 4

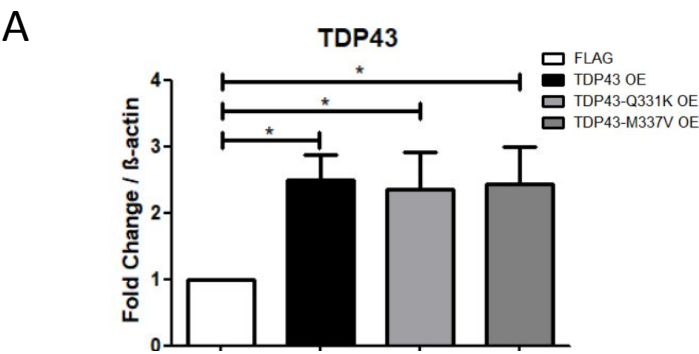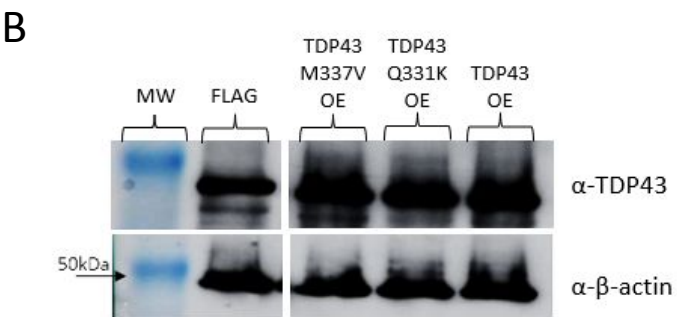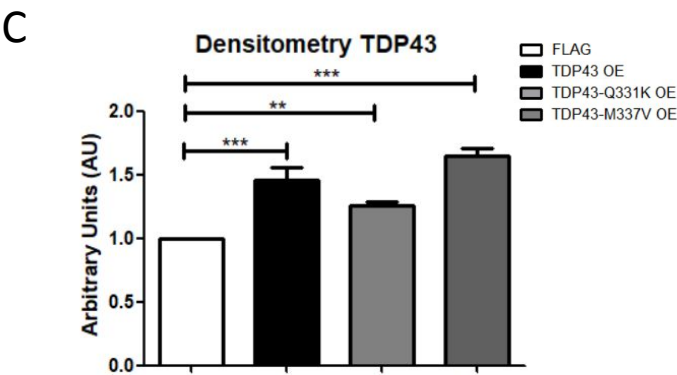

Supplement: Supplementary file 4 — Supporting File 4 [file CBIN-50-0-s005.pdf]

# Supplementary Figure 5

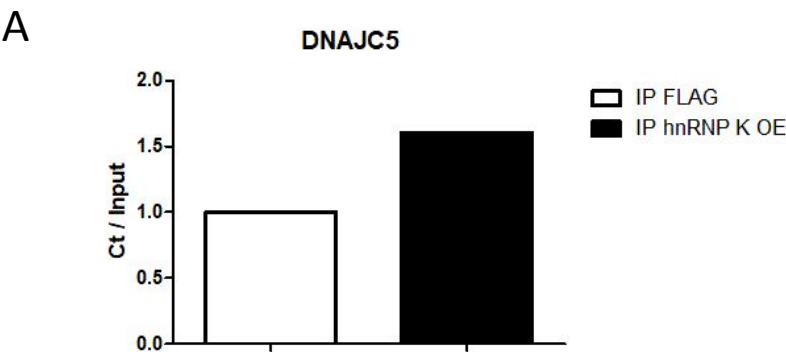

Supplement: Supplementary file 5 — Supporting File 5 [file CBIN-50-0-s003.pdf]

# Supplementary Figure 6

A

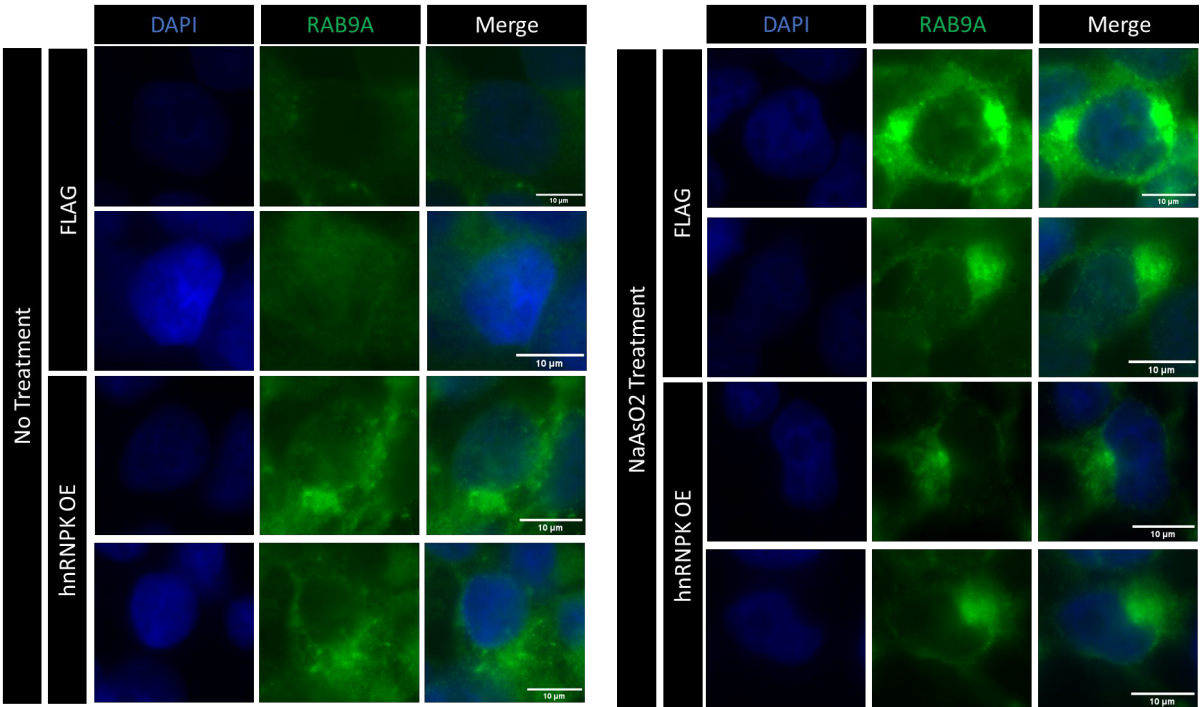

B

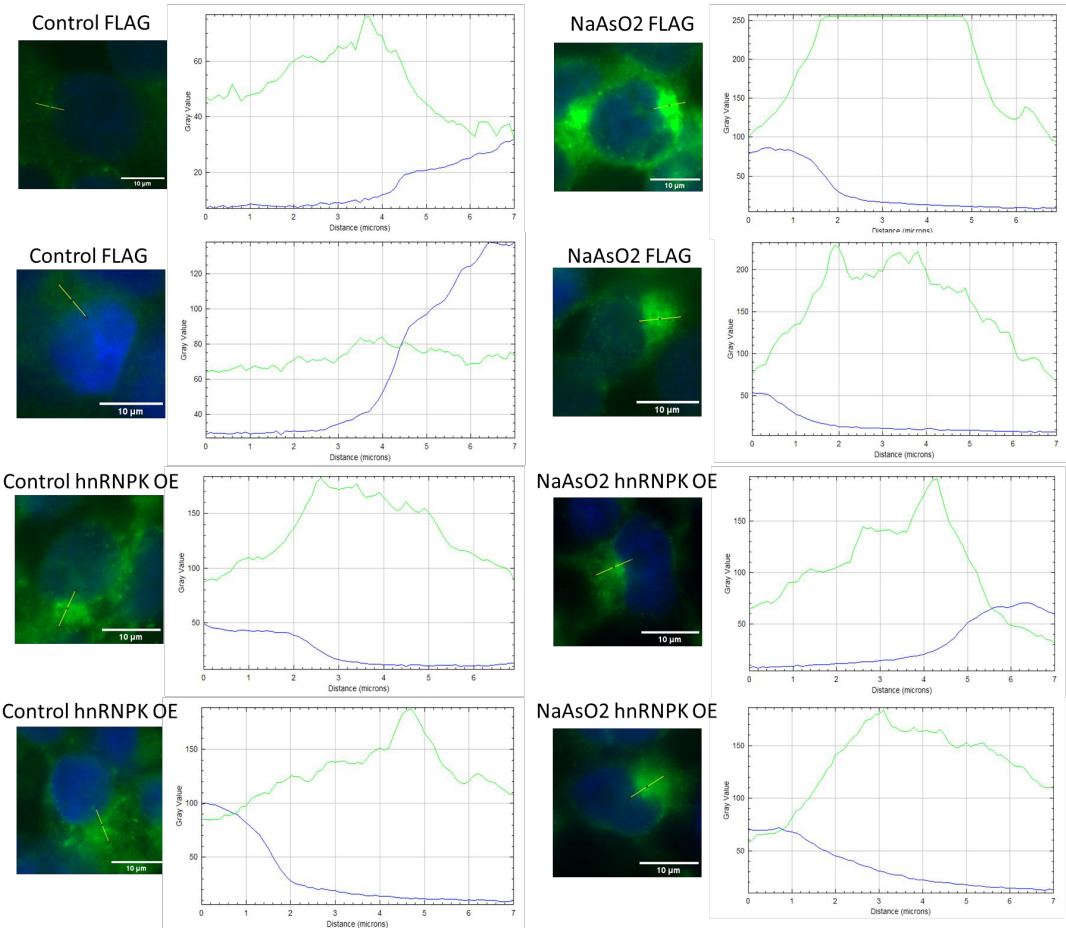

Supplement: Supplementary file 6 — Supporting File 6 [file CBIN-50-0-s001.pdf]
